# ﻿Review of the genus *Paradelius* De Saeger, 1942 of East Asia (Hymenoptera, Braconidae, Cheloninae, Adeliini) with the description of a new species from South Korea

**DOI:** 10.3897/zookeys.1204.123909

**Published:** 2024-06-07

**Authors:** Sergey A. Belokobylskij, Deokseo Ku, Xue-xin Chen

**Affiliations:** 1 Zoological Institute, Russian Academy of Sciences, St Petersburg 199034, Russia Zoological Institute, Russian Academy of Sciences St Petersburg Russia; 2 The Science Museum of Natural Enemies, Geochang 50147, Republic of Korea The Science Museum of Natural Enemies Geochang Republic of Korea; 3 Institute of Insect Sciences, College of Agriculture and Biotechnology, Zhejiang University, Hangzhou 310058, China Zhejiang University Hangzhou China

**Keywords:** Ichneumonoidea, new species, new synonyms, parasitoids, redescriptions, *Sculptomyriola*, *Sinadelius*

## Abstract

The East Palaearctic species of the adeliine genus *Paradelius* De Saeger, 1942 are reviewed. The genus *Sculptomyriola* Belokobylskij, 1988 is synonymised with *Paradelius* and treated as its subgenus. The following species are transferred to subgenus Paradelius (Sculptomyriola): P. (Sc.) extremiorientalis (Belokobylskij, 1988), **comb. nov.**; P. (Sc.) ghilarovi (Belokobylskij, 1988), **comb. nov.**; P. (Sc.) neotropicalis Shimbori & Shaw, 2019; P. (Sc.) nigrus Whitfield, 1988; P. (Sc.) rubrus Whitfield, 1988; P. (Sc.) sinevi (Belokobylskij, 1998), **comb. nov.** A new species Paradelius (Sculptomyriola) koreanus**sp. nov.** from Korean Peninsula is described. The genus *Sinadelius* He & Chen, 2000 is synonymised with Paradelius De Saeger and also treated as its subgenus. The species Sinadeliusguangxiensis He & Chen, 2000 and *S.nigricans* He & Chen, 2000 are transferred to Paradelius (Sinadelius) (**comb. nov.**). A key for determination of the World known *Paradelius* species from three its subgenera, *Paradelius* s.str., *Sculptomyriola* Belokobylskij and *Sinadelius* He & Chen, and illustrated redescriptions of the type of genus and its Asian species are provided.

## ﻿Introduction

The members of the braconid wasps of the small tribe Adeliini have been considered as a separate subfamily in the Braconidae microgasteroid complex for a long time (Čapek 1970; Tobias 1986; Belokobylskij 1988, 1998; Quicke and van Achterberg 1990; van Achterberg 1993; Whitfield 1997; Shi et al. 2005). However, the following morphological and especially molecular phylogenetic analyses of the Braconidae subfamilies (Whitfield and Mason 1994; Dowton and Austin 1998; Kittel et al. 2016) have shown that this taxonomic group is nested within the subfamily Cheloninae, and subsequently it has been treated only as a chelonine tribe (Chen and van Achterberg 2019; Shimbori et al. 2019; Jasso-Martínez et al. 2022).

This subfamily (and later tribe) was long time considered to consist of four genera: *Adelius* Haliday, 1833 (with its synonyms *Acaelius* Haliday, 1834, *Acoelius* Haliday, 1835, *Anomopterus* Rohwer, 1914, *Myriola* Shestakov, 1932, and *Pleiomerus* Wesmael, 1837), *Paradelius* De Saeger, 1942, *Sculptomyriola* Belokobylskij, 1988, and *Sinadelius* He & Chen, 2000 (Yu et al. 2016; Shimbori et al. 2019). The members of this taxonomic group are relatively rarely presented in the scientific collection (perhaps except for *Adelius*), although the adeliine wasps have an almost worldwide distribution.

Adeliine species are known as solitary koinobiont larval or perhaps egg-larval endoparasitoids of mainly leaf-mining moths from predominantly the families Nepti­culidae, but also were reared from the species of the families Coleophoridae, Gracillariidae, Lyonetiidae, Tischeriidae and Tortricidae (Yu et al. 2016; Shimbori et al. 2019).

In this article, the description of a new species, two generic synonyms, five new combinations, and redescriptions of the *Paradelius* type species and seven East Asian species are provided, and a key to all adeliine genera and *Paradelius* species is compiled.

## ﻿Materials and methods

The braconid specimens were examined with an Olympus SZ51 stereomicroscope. Photographs were obtained using a Canon EOS 70D digital camera mounted on an Olympus SZX10 microscope (Zoological Institute RAS, St Petersburg). The photographs of Chinese species were made by a digital microscope (KEYENCE VHX–2000, Osaka, Japan) (Zhejiang University, Hangzhou). Image stacking was performed using Helicon Focus 8.0. The figures were produced using the Adobe Photoshop CS6 and CC2018 programs. In the keys, additional features useful for separating species are listed after the dash (–).

The terminology used for morphological features, sculpture, and body measurements follow Belokobylskij and Maetô (2009). Wing venation nomenclature also follows Belokobylskij and Maetô (2009), with the terminology of van Achterberg (1993) shown in parentheses.

Abbreviations are indicated for the type material as **HT**, holotype and **PT**, paratype. Abbreviations of specimen depositories and collections are as follows:

**AMTB** African Museum, Tervuren, Belgium;

**NIBR**National Institute of Biological Resources, Incheon, Republic of Korea;

**SMNE** the Science Museum of Natural Enemies, Geochang, Republic of Korea;

**ZISP**the Zoological Institute of the Russian Academy of Sciences, St Petersburg, Russia;

**ZJUH**Parasitoid Hymenoptera Collection of the Institute of Insect Sciences, Zhejiang University, Hangzhou, China.

## ﻿Taxonomy

**Class Insecta Linnaeus, 1758**

**Order Hymenoptera Linnaeus, 1758**

**Family Braconidae Nees, 1811**

**Subfamily Cheloninae Foerster, 1863**

**Tribe Adeliini Viereck, 1918**

### ﻿Key to the World genera of the tribe Adeliini

**Table:** 

1	All metasomal tergites smooth, only rarely first tergite shortly rugose-striate in basomedial excavation; sutures between first and second and second and third tergites absent or very weak, present as track	***Adelius* Haliday, 1833**
–	First metasomal tergite entirely, second tergite entirely or at least basally, and often third tergites in basal 0.3–0.8 rugose-reticulate and sometimes partly with striation; sutures between first and second and often also between second and third tergites present, distinct, rather wide and crenulate or rugose, but sometimes partially hidden by irregular sculpture (Figs 1I, 2F, 3F, 5G, 6G, 7G, 9D, 10F, 11G	***Paradelius* De Saeger, 1942 (*Sculptomyriola* Belokobylskij, 1988, syn. nov.; *Sinadelius* He & Chen, 2000, syn. nov.)**

#### ﻿Genus Paradelius

Taxon classificationAnimaliaHymenopteraBraconidae

De Saeger, 1942

536BD4F6-E026-5ECD-9008-0B5FCB0FBF78

Paradelius De Saeger, 1942: 313; Belokobylskij 1988: 148; Whitfield 1988: 313; He et al. 2000: 682; Yu et al. 2016; Shimbori et al. 2019: 190.
Sculptomyriola Belokobylskij, 1988: 145 (type species: S.extremiorientalis Belokobylskij, 1988) (syn. nov.); He et al. 2000: 681; Yu et al. 2016; Shimbori et al. 2019: 154.
Sinadelius He & Chen in He et al. 2000: 681 (type species: S.guangxiensis He & Chen, 2000) (syn. nov.); Yu et al. 2016; Shimbori et al. 2019: 154.

##### Type species.

*Paradeliusghesquierei* De Saeger, 1942, for primary designation and monotypy.

##### Notes.

A careful restudy of the type species of the genus *Paradelius* De Saeger, with the redescribed *P.ghesquierei* De Saeger, as well as the descriptions of all known taxa of this and related genera (Belokobylskij 1988; Whitfield 1988; He et al. 2000; Shimbori et al. 2019) and available material for most Asian *Paradelius* species have shown that the characters suggested for the separation of *Sculptomyriola* from *Paradelius* (mainly fore wing venation, sculpture of metasoma, and condition of prepectal carina) are distinctly variable, not taxonomically stable and as result, not available for use as generic features. The infrageneric variation of the adeliine fore wing venation was already noted in the genus *Adelius* Haliday on the examples of the species originally described in the synonymised genus *Myriola* Shestakov, 1932 (Muesebeck and Walkley 1951; Yu et al. 2016). Additionally, only laterally developed prepectal carina (which is reduced below) in the type species of *Paradelius* De Saeger, *P.ghesquierei* De Saeger, as well as in *P.chinensis* He & Chen, 2000, are variable, weakly visible (often because of distinct sculpture surrounding this carina) or reduced in other species former belonged to *Sculptomyriola*. As a result of this study, *Sculptomyriola* Belokobylskij, 1988 is synonymised under *Paradelius* De Saeger, 1942 (syn. nov.), but we keep this name as a subgeneric subdivision including the larger part of previously described species of *Paradelius*.

The different levels of sculpture covering the first three metasomal tergites found in the *Paradelius* and *Sinadelius* species studied (and even in some *Adelius*) as well as its variation in the sculpture distribution surface, also allow the name *Sinadelius* He & Chen, 2000 to be synonymised with *Paradelius* De Saeger, 1942 (syn. nov.). However, this name with three known eastern Asian species should be kept as a subgeneric subdivision of *Paradelius* because the members of *Sinadelius* have kept sculpture basically only on the whole first and basal part of second tergites (by the way, the second tergite is incompletely sculptured also in the type of the genus, *P.ghesquierei* De Saeger), and with only weakly designated second suture.

##### Redescription of the genus.

Head transverse (dorsal view). Occipital carina distinct and complete, joined below with hypostomal carina. Vertex densely reticulate-punctate, sometimes additionally with irregular transverse striae. Ocelli relatively small, arranged in triangle with base 1.1–1.3× its sides. Eye large, covered by dense and long or short setae. Frons weakly concave, sometimes with medial longitudinal carina. Clypeal suture deep and complete. Clypeus weakly or distinctly convex in lower margin. Malar suture distinct and complete. Antenna relatively short, thickened, setiform or filiform. Scape long and wide, 2.0–2.5× longer than maximum width; pedi­cel short. Flagellar segments in apical quarter of antenna longitudinal or subsquare to transverse. Mesosoma relatively short and high. Mesoscutum densely and distinctly punctate, high and curvedly elevated above pronotum. Notauli absent. Prescutellar depression short or very short and distinctly crenulate. Prepectal carina variable, present laterally at least weakly or completely absent, always absent ventrally. Precoxal sulcus distinct, narrow or wide, long, distinctly sinuate, entirely crenulate or rugulose. Mesopleuron mainly smooth. Propodeum usually with areas delineated by distinct carinae, partly or widely smooth or entirely rugose. Fore wing with large pterostigma. Radial vein (r + 3-SR) with one (r) or two (r + 3-SR) abscissae, with its distal 0.5–0.7 transparent; vein arising from pterostigma separately from first radiomedial vein (2-SR) or joined and from one point of pterostigma, sometimes first radiomedial vein (2-SR) arising from short first abscissa of radial vein (r) closely to pterostigma. Discoidal (discal) cell large, anteriorly usually sessile on parastigma, but sometimes shortly petiolate near parastigma. Recurrent vein (m-cu) usually postfurcal to first radiomedial vein (2-SR), only rarely subinterstitial or very weakly antefurcal. Mediocubital vein (M+CU1) distinctly curved towards longitudinal anal vein (1-1A). Brachial (subdiscal) cell widely open distally; most part of second abscissa of longitudinal anal (2-1A) and brachial (CU1b) veins absent. In hind wing, radial (marginal) cell without additional transverse vein (r). First abscissa of mediocubital vein (M+CU) distinctly longer that its second abscissa (1-M). Nervellus (cu-a) desclerotised and transparent. Hind coxa enlarged; hind femur wide. Hind tibia clavate, distinctly or strongly widened distally; long inner tibial spur not shorter than half of basitarsus. First and second segments of hind tarsus sometimes with distinct transparent keels below. Claw small and distinctly or weakly curved. Metasoma at least weakly depressed dorsoventrally, with immovably fused from first to third tergites, not formed complete coarse carapace and its posterior metasomal tergites usually distinctly protruding behind third tergite. First tergite without or with small dorsope, without dorsal carinae. Both sutures (between first and second, second and third tergites) present, rather deep or shallow, relatively narrow and densely sculptured. First and second tergites entirely or also third tergite at least in basal third or half densely striate-rugulose. Ovipositor short.

##### Composition.

Paradelius (Paradelius) chinensis He & Chen, 2000, P. (P.) ghesquierei De Saeger, 1942; P. (Sculptomyriola) extremiorientalis (Belokobylskij, 1988), comb. nov., P. (Sc.) ghilarovi (Belokobylskij, 1988), comb. nov., P. (Sc.) koreanus Belokobylskij & Ku, sp. nov., P. (Sc.) neotropicalis Shimbori & Shaw, 2019, P. (Sc.) nigrus Whitfield, 1988, P. (Sc.) rubrus Whitfield, 1988, P. (Sc.) sinevi (Belokobylskij, 1998), comb. nov.; P. (Sinadelius) guangxiensis (He & Chen, 2000), comb. nov., P. (S.) nigricans (He & Chen, 2000), comb. nov., P. (S.) ussuriensis Belokobylskij, 1988.

##### Hosts.

Solitary koinobiont endoparasitoids of leaf-mining moths, *Stigmella* sp. and *Stigmellavariella* (Braun, 1910) (Nepticulidae), as well as *Enarmonia* sp. (Tortricidae) (Lepidoptera) (Yu et al. 2016; Shimbori et al. 2019).

A host record for *P.ghesquierei*, *Enarmonia* sp. (Tortricidae) (De Saeger 1942), has not been confirmed later and might be erroneous (Shimbori et al. 2019).

##### Distribution.

East Palaearctic (Russia: Far East; China: Liaoning; Korean Peninsula), Oriental (China: Zhejiang, Guangxi); Afrotropics (D.R. Congo); Nearctic (USA); Neotropics (Costa Rica).

### ﻿Key to the World species of the genus *Paradelius* De Saeger

**Table:** 

1	Prepectal carina present laterally and rather distinctly visible. – Propodeum mainly smooth between carinae of areas(Figs 1H, 2F). Third metasomal tergite entirely smooth (Figs 1I, 2F). (Subgenus Paradelius s. str.)	**2**
–	Prepectal carina laterally usually absent or very weakly visible	**3**
2	Recurrent vein (m-cu) of fore wing postfurcal to first radiomedial vein (3-SR) (Figs 1K, 4C). Head behind eyes weakly convex in anterior half and roundly narrowed in posterior half; transverse diameter of eye almost twice longer than temple (dorsal view) (Fig. 1D). Second metasomal tergite rugose-striate in basal 0.8, smooth posteriorly. – D.R. Congo	**P. (P.) ghesquierei De Saeger, 1942**
–	Recurrent vein (m-cu) of fore wing very weakly antefurcal to first radiomedial vein (3-SR) (Figs 2H, 4A). Head behind eyes entirely distinctly roundly narrowed; transverse diameter of eye ~ 4.5× longer than temple (dorsal view) (Fig. 2C). Second metasomal tergite entirely striate with rugosity. – China (Zhejiang)	**P. (P.) chinensis He & Chen, 2000**
3	First (between first and second tergites) and second (between second and third tergites) sutures distinct and relatively wide. First and second tergites entirely, and often third tergite in basal 0.3–0.8 rugose-reticulate or striate with reticulation (Figs 1I, 2F, 3F, 5G, 6G, 7G). Antenna 20-segmented (Subgenus Sculptomyriola Belokobylskij, 1988)	**4**
–	Only first suture (between first and second tergites) distinct and relatively wide; second suture very fine. First tergite entirely and second tergite only basally rugose-reticulate (Figs 9D, 10F, 11G). Antenna 22–23-segmented (Subgenus Sinadelius He & Chen, 2000)	**11**
4	Radial vein (r) of fore wing only with single abscissa; first radiomedial vein (2-SR) arising from pterostigma usually distant from radial vein (r), or rarely from one point with radial vein (r) (Fig. 4). Medial vein (1-SR+M) of fore wing arising from basal vein (1-M) relatively far from parastigma; discoidal (discal) cell distinctly petiolate or subpetiolate anteriorly (Figs 3H, 4C)	**5**
–	Radial vein of fore wing with two abscissae (r and 3-SR), first abscissa (r) short; first radiomedial vein (2-SR) arising from radial vein (r) (Fig. 8). Medial vein (1-SR+M) of fore wing arising from parastigma; discoidal (discal) cell sessile anteriorly (Fig. 8)	**6**
5	First radiomedial vein (2-SR) arising from pterostigma from one point with radial vein (r). Sclerotised basal part of metacarp (1-R1) 0.4–0.5× as long as pterostigma (Figs 3H, 4C). Tenth–twentieth antennal segments subsquare (Fig. 3D). Body usually entirely black, rarely head partly reddish brown (Fig. 3A). – Russia (south of Far East), Korean Peninsula (See also couplet 8)	**P. (Sc.) extremiorientalis (Belokobylskij, 1988), comb. nov.**
–	First radiomedial vein (2-SR) arising from pterostigma distant from radial vein (r). Sclerotised basal part of metacarp (1-R1) 0.2–0.3× as long as pterostigma. Tenth–twentieth antennal segments weakly elongate. Body entirely or at least partly reddish brown, pale reddish brown or yellowish brown. – USA (California, Wyoming)	**P. (Sc.) rubrus Whitfield, 1988**
6	Body completely brownish yellow (Fig. 6A). Third metasomal tergites mainly smooth, only finely rugulose in narrow basomedial part (Fig. 6G). Small, body length 1.5 mm. – Medial segments of antenna weakly transverse or subsquare (Fig. 6D). Propodeum with long and inversely-pentagonal areola delineated by strong and complete carinae (Fig. 6G). First and second metasomal sutures rather weak (Fig. 6G). – Korean Peninsula	**P. (Sc.) koreanus sp. nov.**
–	Body completely dark brown or black, only sometimes with reddish brown areas on head and anterior parts of mesosoma (Figs 5A, 7A). Third metasomal tergites mainly rugose-reticulate and sometimes with additional striation, smooth only in distal 0.2–0.5 (Figs 5G, 7G). Large, body length 2.1–2.7 mm	**7**
7	Antennae distinctly shorter than body, in female length of segments behind middle of antenna distinctly less than their width, these segments transverse (Fig. 5D). Penultimate segment of female antenna 0.75–0.85× as long as wide (Fig. 5D). Body only partly black, most part of head, prothorax, mesoscutum in anterior 0.3–0.5 and part or all mesopleuron reddish brown (Fig. 5A). – Russia (south of Far East), Korean Peninsula	**P. (Sc.) ghilarovi (Belokobylskij, 1988), comb. nov.**
–	Antennae almost equal to or longer than body, in female length of segments behind middle of antenna not less than their width, these segments subsquare or elongated (Fig. 7D). Penultimate segment of female antenna 1.3–1.5× longer than wide (Fig. 7D) (unknown in *P.nigra* and *P.neotropicalis*). Body completely black (Fig. 7A), very rarely head partly (dark) reddish brown	**8**
8	Medial vein (1-SR+M) of fore wing arising from basal vein (1-M) relatively far from parastigma; discoidal (discal) cell distinctly petiolate anteriorly (Figs 3H, 4B). Sclerotised basal part of metacarp (1-R1) 0.4–0.5× as long as pterostigma (Figs 3H, 4B). – Tenth–twentieth antennal segments subsquare (Fig. 3D). (See also couplet 5)	**P. (Sc.) extremiorientalis (Belokobylskij, 1988), comb. nov.**
–	Medial vein (1-SR+M) of fore wing arising from parastigma; discoidal (discal) cell sessile anteriorly (Figs 7H, 8C). Sclerotised basal part of metacarp (1-R1) 0.2–0.3× as long as pterostigma (Figs 7H, 8C)	**9**
9	Sutures between first and second and second and third tergites distinct and relatively wide (Fig. 3FG). Propodeum smooth within carinae of areas. – USA (Texas)	**P. (Sc.) nigrus Whitfield, 1988**
–	Sutures between first and second and second and third tergites rather weak and poorly visible, covered by additional sculpture (Fig. 7G). Propodeum rugose-reticulate within distinct carinae of areas (Fig. 7G)	**10**
10	Fore wing entirely hyaline (Figs 7H, 8C). Antenna basally pale reddish brown (Fig. 7D). All legs mainly pale reddish brown or yellowish brown (Fig. 7A). Third metasomal tergite rugose-reticulate in basal 0.7–0.8 (Fig. 7G). – Russia (south of Far East)	**P. (Sc.) sinevi (Belokobylskij, 1998), comb. nov.**
–	Fore wing medially distinctly and widely infuscate. Antenna basally dark reddish brown to black. All legs mainly dark reddish brown to black. Third metasomal tergite longitudinally striate in basal 0.8–0.9. – Costa Rica	**P. (Sc.) neotropicalis Shimbori & Shaw, 2019**
11	Body mainly yellowish brown (Fig. 9A). Transverse diameter of eye ~ 2.5× larger than length of temple (dorsal view) (Fig. 9C). First metasomal tergite behind spiracular tubercles subparallel-sided (Fig. 9D). – China (Guangxi)	**P. (S.) guangxiensis (He & Chen, 2000), comb. nov.**
–	Body mainly black or dark brown, however metasoma often reddish brown (Figs 10A, 11A). Transverse diameter of eye 1.2–1.5× larger than length of temple (dorsal view) (Figs 10C, 11C). First metasomal tergite behind spiracular tubercles narrowed towards its apex (Figs 10F, 11G)	**12**
12	First metasomal tergite behind spiracular tubercles distinctly narrowed towards its apex (Fig. 11G). Second suture poorly visible (Fig. 11G). – Russia (Primorskiy Territory)	**P. (*S.*) *ussuriensis* Belokobylskij, 1988**
–	First metasomal tergite behind spiracular tubercles weakly narrowed towards its apex (Fig. 10F). Second suture distinct (Fig. 10F). – China (Liaoning)	**P. (*S.*) *nigricans* (He & Chen, 2000), comb. nov.**

**Subgenus Paradelius s. str.**

Figs 1, 2

#### ﻿ Paradelius (Paradelius) ghesquierei

Taxon classificationAnimaliaHymenopteraBraconidae

De Saeger, 1942

C99F0AEF-354A-5B73-9C96-A7ABD730CD6F

Figs 1 , 4A

Paradelius ghesquierei De Saeger 1942: 314; Belokobylskij 1988: 148; Whitfield 1988: 313; Yu et al. 2016.

##### Material examined.

D.R. of Congo: “Holotypus *P.ghesquierei* D.S. ♂ [Sic!]” (red), “Musée du Congo, 3728, Rutshuru, II – 1937, J. Ghesquière”, “♀”, “R. dét. X. 4690”, “H. De Saeger det. 1942: *Paradelius Ghesquierei.* Holotype ♀”, 1 female (HT) (AMTB).

##### Description.

**Female**. Body length 2.0 mm; fore wing length 1.8 mm.

***Head***. Head almost twice wider than its medial length (dorsal view), 1.2× wider than mesoscutum. Occiput distinctly evenly concave. Head behind eyes weakly convex in anterior half and roundly narrowed in posterior half; transverse diameter of eye almost twice larger than length of temple (dorsal view). Ocelli arranged in equilateral triangle. POL 1.3× Od, 0.4× OOL. Eye 1.3× as high as broad. Malar space almost equal to basal width of mandible, 0.2× height of eye. Face convex, width of face 1.3× its median height, 0.9× height of eye. Tentorial pits small, distance between pits 1.25× distance from pit to eye. Clypeus high and weakly convex, its width 1.8× median height, 0.8× width of face; its lower margin convex medially. Head strongly roundly narrowed below eyes (front view).

**Figure 1.**
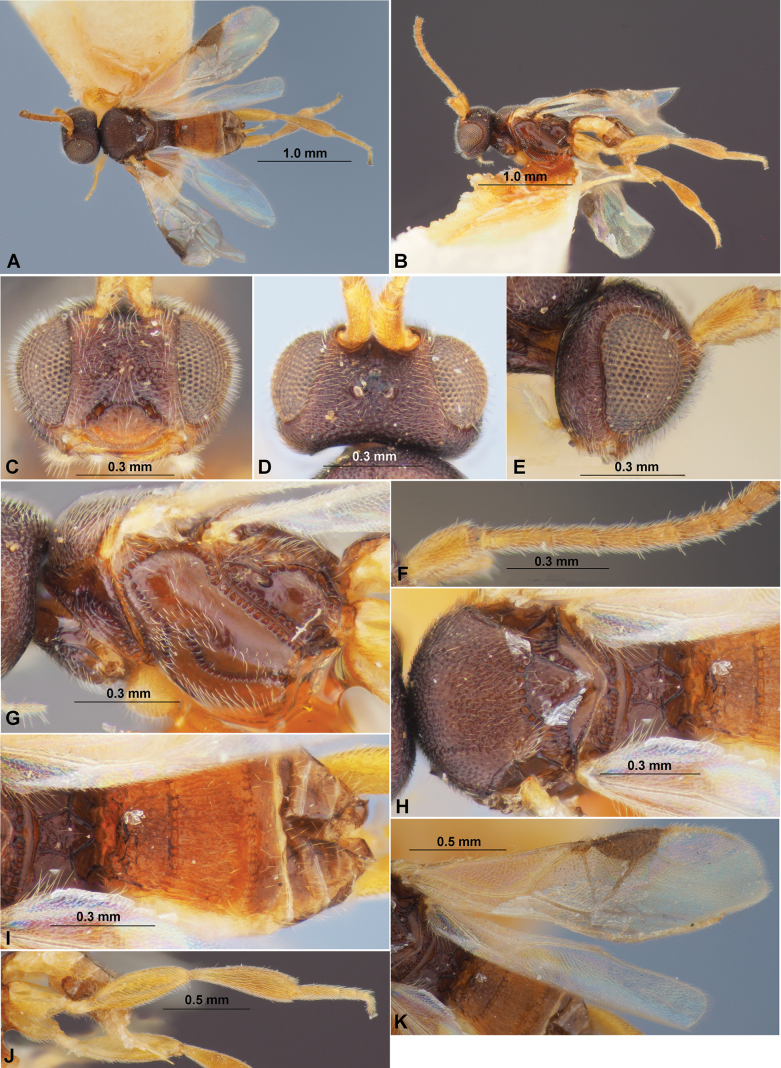
Paradelius (Paradelius) ghesquierei De Saeger, 1942 (female, holotype) **A** habitus, dorsal view **B** habitus, lateral view **C** head, front view **D** head, dorsal view **E** head, lateral view **F** antenna, basal segments **G** mesosoma, lateral view **H** mesosoma, dorsal view **I** metasoma, dorsal view **J** hind leg **K** wings.

***Antenna***. Antenna partly missing apically, 20-segmented (according to original description), weakly thickened, all preserved submedial segments elongated, but 10^th^ segment shorter and wide. Scape 2.2× longer than wide. First flagellar segment 3.8× longer than its apical width, 1.5× longer than second segment. Tenth antennal segment 1.3× as long as its width.

***Mesosoma***. Mesosoma 1.4× longer than maximum height. Mesoscutum highly and curvedly elevated above pronotum (lateral view), 1.6× wider than its medial length (dorsal view). Prescutellar depression (scutellar sulcus) short and shallow, with numerous distinct carinae. Scutellum 1.1× longer than anterior width. Prepectal carina present laterally and absent ventrally. Precoxal sulcus distinct, long, oblique, sinuate, extending below almost throughout all mesopleuron, crenulate.

***Wings***. Fore wing 3.2× longer than maximum width. Pterostigma 2.7× longer than its maximum width. Radial vein (r) arising from distal 0.2 of pterostigma and far from point of arising of first radiomedial vein (2-SR). Present only single abscissa of radial vein (r), which distinctly curved, almost entirely desclerotised and reaching as track distal margin of wing. Radial (marginal) cell shortened, 2.5× longer than its maximum width. Metacarp (1-R1) sclerotised at most basal part, its sclerotised part 1.2× longer than pterostigma. First radiomedial vein (2-SR) mainly distinctly sclerotised, almost 5.0× longer than recurrent vein (m-cu). Recurrent vein (m-cu) subinterstitial to first radiomedial vein (2-SR), posteriorly weakly convergent with basal vein (1-M). Discoidal (discal) cell widely sessile anteriorly, 1.2× longer than its maximum width. Nervulus (cu-a) subperpendi­cular to longitudinal anal vein (1-1A), weakly postfurcal, distance between basal vein (1-M) and nervulus (cu-a) 0.25× nervulus (cu-a) length. Hind wing 3.3× longer than maximum width. First abscissa of mediocubital vein (M+CU) almost 2.0× longer than second abscissa (1-M).

***Legs***. Hind coxa long, ~ 2.0× longer than maximum width, 1.5× longer than propodeum (lateral view). Hind femur 3.2× longer than maximum width. Hind tibia claviform, 3.9× longer than maximum width, only weakly narrower than hind femur; longest inner tibial spur 0.6× length of hind basitarsus. Hind tarsus as long as hind tibia, its basitarsus 0.7× as long as second–fifth segments combined, 2.5× longer than second segment, 3.3× longer than fifth segments (without pretarsus).

***Metasoma***. Metasoma almost as long as mesosoma. First to third tergites distinctly sclerotised, with distinct, narrow, curved, complete and crenulate first and second sutures; following tergites relatively weak sclerotised. Medial length of first tergite 0.6× its apical width, as long as second tergite. Second tergite 2.5× longer than third tergite. Length of first to third tergites combined 0.8× their maximum width. Third tergite almost straight on posterior margin. Ovipositor sheaths narrow, rather short, ~ 0.5× as long as first–third tergites combined.

***Sculpture***. Head densely areolate-punctate, clypeus weakly and sparsely punctate, smooth between punctures. Mesoscutum densely and distinctly punctate; scutellum mainly smooth. Mesopleuron mainly smooth, only partly with fine and sparse punctation; metapleuron smooth. Propodeum with areas distinctly delineated by carinae, with large and inversely-pentagonal areola, elongated anterolateral and transverse posterolateral areas, petiolate area short, subsquare; propodeum mainly rugulose between carinae. First tergite entirely and second tergite mostly (in basal 0.8) distinctly and densely rugose-striate, first tergite basally only rugose; third and following tergites smooth.

***Colour***. Body mainly reddish brown to dark reddish brown partly, first to third tergites yellowish brown. Antenna pale brown, brownish yellow in basal third. Palpi pale yellow. Legs mainly yellow, distally brownish yellow. Fore wing subhyaline basally and apically, with faintly infuscate band under pterostigma. Pterostigma and parastigma dark brown, most veins yellow.

**Male**. Unknown.

##### Distribution.

Democratic Republic of Congo.

##### Host.

Undetermined species of *Enarmonia* Hübner, 1825 (Lepidoptera: Tortricidae) on *Zehneriascabra* (Linn. f.) Sond (Cucurbitaceae) (De Saeger, 1942).

#### ﻿ Paradelius (Paradelius) chinensis

Taxon classificationAnimaliaHymenopteraBraconidae

He & Chen, 2000

4453B908-B7F4-56D6-9CA8-5E78C1557BCC

Figs 2 , 4B

Paradelius chinensis He & Chen in He et al. 2000: 682; Yu et al. 2016.

##### Material examined.

China: Zhejiang Province, Qingyuan, Mt. Baishanzu, 30.IX.1993 (Wu Hong col.), No. 941501, 1 female (HT) (ZJUH).

##### Redescription.

**Female**. Body length 1.8 mm; fore wing length 1.6 mm.

***Head***. Head 1.9× wider than its medial length (dorsal view), 1.1× wider than mesoscutum. Occiput weakly evenly concave. Head behind eyes distinctly roundly narrowed; transverse diameter of eye ~ 4.5× larger than length of temple in dorsal view or ~ 2.0× in lateral view. Ocelli arranged in triangle with base 1.2× its sides. POL 1.4× Od, 0.5× OOL. Eye 1.7× as high as broad. Malar space almost equal to basal width of mandible, 0.15× height of eye. Face weakly convex, width of face ~ 1.2× its median height. Tentorial pits small, distance between pits 1.4× distance from pit to eye. Clypeus high and weakly convex, its width 2.5× median height, 0.8× width of face; its lower margin medially almost straight. Head strongly roundly narrowed below eyes (front view).

**Figure 2.**
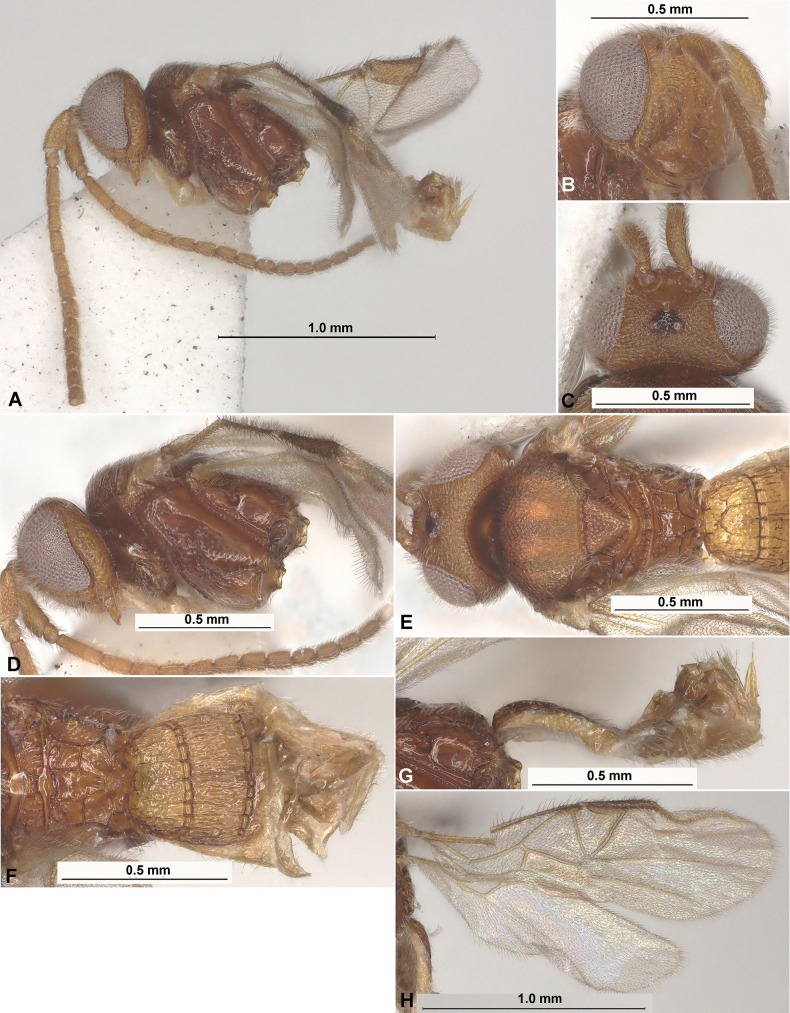
Paradelius (Paradelius) chinensis He & Chen, 2000 (female, holotype) **A** habitus, lateral view **B** head, latero-frontal view **C** head, dorsal view **D** head and mesosoma, lateral view **E** head, mesosoma and base of metasoma, dorsal view **F** propodeum and metasoma, dorsal view **G** propodeum and metasoma, lateral view **H** wings.

***Antenna***. Antenna 20-segmented, thickened, weakly setiform, submedial and apical segments elongate. Scape 2.1× longer than wide. First flagellar segment 3.7× longer than its apical width, 1.6× longer than second segment. Tenth segment elongate, 1.6× longer than wide.

***Mesosoma***. Mesosoma 1.4× longer than maximum height. Mesoscutum highly and curvedly elevated above pronotum (lateral view), 1.7× as wide as its medial length (dorsal view). Prescutellar depression (scutellar sulcus) short and shallow, with numerous distinct carinae. Scutellum 0.8× as long as anterior width. Prepectal carina present laterally and absent ventrally. Precoxal sulcus distinct, long, oblique, sinuate, extending below almost throughout all mesopleuron, crenulate-rugose.

***Wings***. Fore wing damaged and distinctly dented anteriorly, ~ 3.0× longer than maximum width. Radial vein (r) arising almost from distal 0.3 of pterostigma and far from first radiomedial vein (2-SR). Present only single abscissa of radial vein (r), this vein distinctly curved, mainly desclerotised and reaching as track distal margin of wing. Radial (marginal) cell shortened. Metacarp (1-R1) sclerotised at basal part, its sclerotised part ~ 0.6× as long as pterostigma. First radiomedial vein (2-SR) distinctly sclerotised, ~ 6.0× longer than recurrent vein (m-cu). Recurrent vein (m-cu) very weakly antefurcal to first radiomedial vein (2-SR), posteriorly weakly divergent with basal vein (1-M). Discoidal (discal) cell broadly sessile anteriorly, 1.4× longer than its maximum width. Nervulus (cu-a) perpendicular to longitudinal anal vein (1-1A), postfurcal, distance between basal vein (1-M) and nervulus (cu-a) 0.5× nervulus (cu-a) length. Hind wing ~ 3.0× longer than maximum width. First abscissa of mediocubital vein (M+CU) ~ 2.7× longer than second abscissa (1-M).

***Legs***. Legs entirely missing.

***Metasoma***. Metasoma 1.1× longer than mesosoma. First to third tergites sclerotised, with distinct, relatively wide, sparsely crenulate and weakly curved first and second sutures; following tergites relatively weakly sclerotised. Medial length of first tergite 0.6× its apical width, approximately as long as second tergite. Second tergite almost 2.0× longer than short third tergite. Length of first to third tergites combined 1.15× their maximum width. Third tergite weakly curved in posterior margin. Ovipositor sheath narrow and short, ~ 0.25× as long as first–third tergites combined.

***Sculpture***. Vertex densely and small areolate-punctate; frons areolate-punctate, but almost smooth medially; face transverse striate with reticulation between striae, only rugulose-reticulate medially, clypeus rather weakly and sparsely punctate, smooth between punctures. Mesoscutum densely punctate, with dense striation in medio-posterior one–third; scutellum rather densely and distinctly punctate, smooth between punctures. Mesopleuron smooth in long posterior area, mainly punctate with reticulation in anterior lower two–thirds; metapleuron mainly smooth, reticulate-areolate marginally. Propodeum mainly widely smooth, with areas distinctly delineated by high carinae, with wide and inversely-pentagonal areola (wide anteriorly and narrow posteriorly), anterolateral areas subsquare, posterolateral areas transverse-curved. First and second tergites entirely and sparsely striate, with dense rugosity between striae. Third and following tergites smooth.

***Colour***. Body mainly pale reddish brown, mesosoma reddish brown. Antenna yellowish brown. Palpi pale yellow. Fore coxa pale brown. Fore wing entirely faintly infuscate, without dark band under pterostigma. Pterostigma and parastigma brown; most veins brown.

**Male**. Unknown.

##### Distribution.

China (Zhejiang).

#### ﻿ Subgenus Sculptomyriola

Taxon classificationAnimaliaHymenopteraBraconidae

Belokobylskij, 1988

0167D0ED-F1DA-5936-ACA7-DCCB48BA6A57

**Figure 3.**
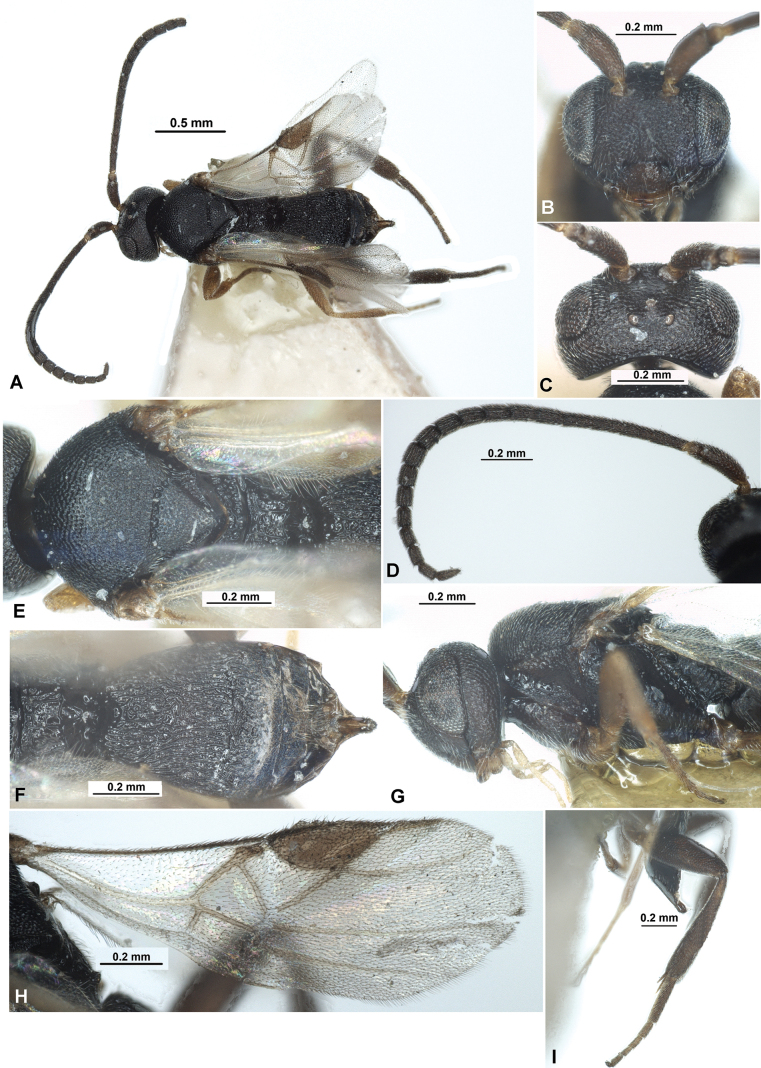
Paradelius (Sculptomyriola) extremiorientalis (Belokobylskij, 1988), comb. nov. (female, holotype) **A** habitus, dorsal view **B** head, front view **C** head, dorsal view **D** antenna **E** mesosoma, dorsal view **F** propodeum and metasoma, dorsal view **G** head and mesosoma, lateral view **H** wings **I** hind leg.

Figs 3 , 5 , 6 , 7 , 8

##### Description of subgenus.

Vertex densely and coarsely reticulate-rugulose. Ocelli arranged in weakly obtuse or subequilateral triangle. Eye covered by dense and relatively long setae. Antenna long, thickened and setiform. Flagellar segments longitudinal, subsquare or weakly transverse in apical half of antenna. Mesoscutum densely and distinctly punctate. Prescutellar depression short and densely crenulate. Prepectal carina laterally weakly present or completely absent. Precoxal sulcus distinct, long, relatively narrow, sinuate, entirely crenulate or rugulose. Propodeum widely rugulose-reticulate, rarely also with areas delineated by carinae at least posteriorly. Fore wing with large pterostigma. Radial vein (r + 3-SR) with one (r) or two (r + 3-SR) abscissae, arising from posterior 0.3 of pterostigma separately or joined with first radiomedial vein (2-SR). Discoidal (discal) cell anteriorly usually sessile on parastigma, but sometimes shortly petiolate. Recurrent vein (m-cu) usually postfurcal to first radiomedial vein (2-SR), only rarely subinterstitial or very weakly antefurcal. Hind femur wide; hind tibia distinctly clavate. First tergite of metasoma distinctly widened towards apex. Both first metasomal sutures present, rather deep and relatively distinct, densely sculptured. First and second tergites entirely and also third tergite at least in basal third or half densely striate-rugulose. Ovipositor short.

#### ﻿ Paradelius (Sculptomyriola) extremiorientalis

Taxon classificationAnimaliaHymenopteraBraconidae

(Belokobylskij, 1988) comb. nov.

5CEF3EAB-1E9D-53BA-9E30-CAD5F58AC0C8

Figs 3 , 4C

Sculptomyriola extremiorientalis Belokobylskij, 1988: 145; 1998: 554; Ku et al. 2001: 15; Yu et al. 2016.

##### Material examined.

Russia. Primorskiy Territory: “Primorskiy Territory, [Partizansk District], env. Sergeevka, Belokobylskij [col.], forest, 21.VII.1979”, “Holotypus *Sculptomyriolaextremiorientalis* Belokobylsk.[ij]”, 1 female (HT) (ZISP); ‘Kedrovaya Pad’ Nature Reserve, 12.VIII.1976 (Berezantsev), 1 male (PT); 30 km S of Slavyanka, at light, 2, 3 & 5.VIII.1985 (S. Belokobylskij), 5 females (PTs); same locality, forest, clearings, 5.VIII.1985 (S. Belokobylskij), 2 females (PTs); 25 km S of Slavyanka, Vityaz’, at light, 2.VIII.1982 (I. Kerzhner), 3 females; Vladivostok, Sedanka, forest, 31.VII.1984 (S. Belokobylskij), 1 female (PT); 10 km S of Partizansk, forest, 18.VII.1979 (S. Belokobylskij), 1 female (PT); 15 km SE of Partizansk, oak-forest, 22.VII.1984 (S. Belokobylskij), 10 females, 2 males (PTs); 10 km SE of Partizansk, oak-forest, 22.VII.1984 (S. Belokobylskij), 2 females; Lazovskiy Nature Reserve, 10 km SW of Sokolchi, rocks, mixed forest, 23.VII.1993 (S. Belokobylskij), 1 female, 1 male; 20 km N of Rudnaya Pristan’, oak-forest, 18.VII.1979 (S. Belokobylskij), 1 female (PT); 20 km SE of Ussuriysk, at light, 31.VII & 1–4.VIII.1991 (S. Belokobylskij), 4 females, 1 male; same locality, forest, clearings, 1 & 5.VIII.1991 (S. Belokobylskij), 3 females; 20 km E of Ussuriysk, at light, 26.VII.1999 (S. Sinev), 1 female; 25 km E of Spassk-Dal’niy, forest, 12.VII.1991 (S. Belokobylskij), 1 female; 20 km SE of Spassk-Dal’niy, Evseevka, forest, 18.VII.1991 (S. Belokobylskij), 1 female; 20 km NNW of Spassk-Dal’niy, Novosel’skoe, meadow, bush, 19.VII.1998 (S. Belokobylskij), 1 female; Spassk-Dal’niy, forest, 13.VII.1991 (S. Belokobylskij), 1 female. Kuril Islands: Shikotan I., 5–7 km S of Krabozavodsk, 17.VIII.1973 (D. Kasparyan), 1 male (PT) (All in ZISP).

**Figure 4.**
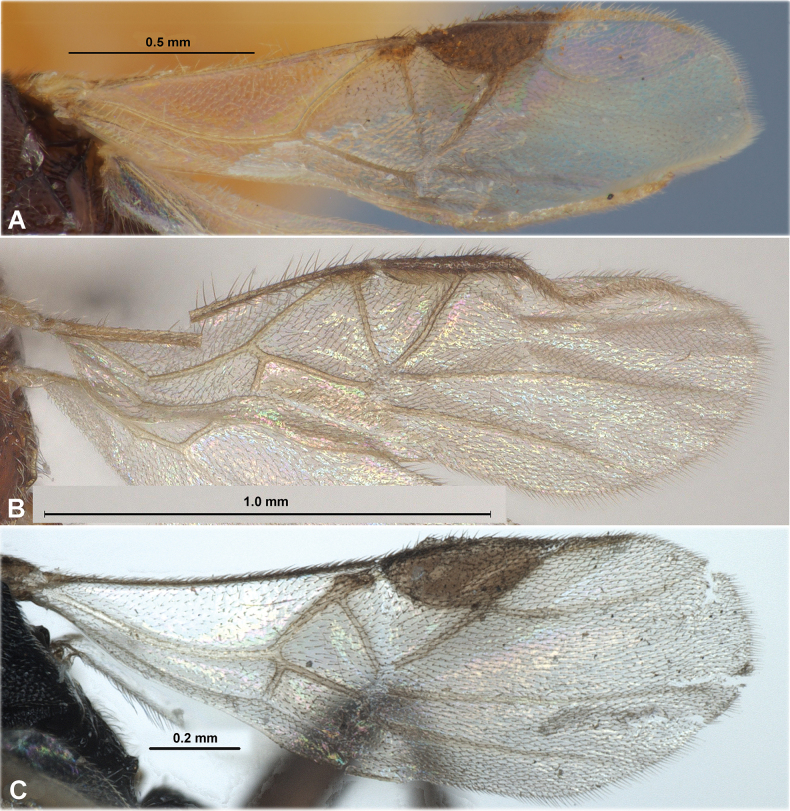
Fore wings. **A**Paradelius (Paradelius) ghesquierei De Saeger **B**P. (P.) chinensis He & Chen **C**P. (Sculptomyriola) extremiorientalis (Belokobylskij).

South Korea. Gyeonggi-do, Mt. Chungnyeong, at light, 3.VII–16.VIII.1999 (D. Ku), 1 female (SMNE); Gyeongsangnam-do, Geochang-gun, Sinwon-myeon, Waryong-ri, Malaise Trap, 18.VI–2.VII.2022 (D. Ku, J. Lee, H. Jeong), 1 female (SMNE); Gyeongsangnam-do, Goseong-gun, Hail-myeon, Suyang-ri, 34°58'34.8"N, 128°12'08.3"E, 23.VI.2023 (S. Belokobylskij), 1 male (ZISP); Gyeongsangnam-do, Sancheong-gun, 30 km NNW of Jinju, forest, bush, 800 m, 29.VI.2002 (S. Belokobylskij), 1 female (ZISP).

##### Description.

**Female**. Body length 2.0–2.6 mm; fore wing length 1.7–2.0 mm.

***Head***. Head 2.0–2.2× wider than its medial length (dorsal view), approxi­mately as wide as mesoscutum. Occiput distinctly evenly concave. Head behind eyes weakly convex to roundly narrowed; transverse diameter of eye 1.2–1.4× larger than length of temple (dorsal view). Ocelli arranged in triangle with base 1.2–1.3× its sides. POL 2.0–2.5× Od, 0.6–0.7× OOL. Eye 1.5–1.6× as high as broad. Malar space 1.0–1.2× basal width of mandible, 0.2–0.3× height of eye. Face convex, width of face 1.6–1.7× its median height, 1.1–1.3× height of eye. Tentorial pits small, distance between pits 1.1–1.3× distance from pit to eye. Clypeus high and weakly convex, its width 1.5–1.7× median height, ~ 0.6× width of face. Head strongly roundly narrowed below eyes (front view).

***Antenna.*** Antenna 20-segmented, thickened, setiform, its apical segments elongate, but medial ones often subsquare. Scape 2.2–2.6× longer than wide. First flagellar segment 2.6–2.8× longer than its apical width, 1.1–1.2× longer than second segment. Tenth–twentieth segments subsquare. Penultimate segment 1.1–1.5× longer than its width, 0.7–0.8× as long as obtuse apical segment.

***Mesosoma***. Mesosoma 1.5–1.7× longer than maximum height. Mesoscutum highly and curvedly elevated above pronotum (lateral view), 1.4–1.5× as wide as its medial length (dorsal view). Prescutellar depression (scutellar sulcus) shallow, with distinct numerous carinae. Scutellum 0.7–0.8× as long as anterior width. Prepectal carina widely absent, sometimes only weakly or very weakly visible laterally. Precoxal sulcus distinct, oblique, extending below almost throughout all lower part of mesopleuron, rugulose.

***Wings***. Fore wing 2.6–2.8× longer than maximum width. Pterostigma 2.5–2.7× longer than its maximum width. Radial vein (r) arising from distal 0.4 of pterostigma. First radiomedial vein (2-SR) arising from pterostigma from one point with radial vein (r) or (more usually) weakly distant from it. Present two abscissae of radial vein (r and 3-SR) with very short first radial abscissa (r) and long second abscissa (3-SR), but sometimes present only single abscissa of radial vein (r); second abscissa (3-SR) weakly curved, sclerotised in basal 0.2–0.3 and desclerotised on remaining part, reaching as track distal margin of wing. Radial (marginal) cell shortened, 2.5–4.0× longer than its maximum width. Metacarp (1-R1) sclerotised in basal half, its sclerotised part 0.4–0.5× as long as pterostigma. First radiomedial vein (2-SR) mainly distinctly sclerotised, 4.0–6.0× longer than recurrent vein (m-cu). Recurrent vein (m-cu) distinctly postfurcal to first radiomedial vein (2-SR), posteriorly subparallel with basal vein (1-M). Discoidal (discal) cell shortly petiolate anteriorly 1.3–1.4× longer than its maximum width. Nervulus (cu-a) subperpendicular to longitudinal anal vein (1-1A), weakly postfurcal, distance between basal vein (1-M) and nervulus (cu-a) ~ 0.3× nervulus (cu-a) length. Hind wing 3.2–3.4× longer than maximum width. First abscissa of mediocubital vein (M+CU) 2.0–2.5× longer than second abscissa (1-M).

***Legs***. Hind coxa long, 1.7–1.8× longer than maximum width, 1.4–1.5× longer than propodeum (lateral view). Hind femur 3.1–3.5× longer than maximum width. Hind tibia claviform, 4.5–5.2× longer than maximum width, 0.7–0.8× as wide as hind femur; longest inner tibial spur ~ 0.5× hind basitarsus length. Hind tarsus as long as hind tibia, its basitarsus 0.8–0.9× as long as second–fifth segments combined, 2.5–3.0× longer than second segment, 3.3–3.5× longer than fifth segment (without pretarsus).

***Metasoma***. Metasoma 0.8–0.9× as long as mesosoma. All tergites (especially first to third ones) distinctly sclerotised. First suture shallow, poorly visible, strongly curved; second suture distinct, narrow, weakly curved. Medial length of first tergite 0.6–0.7× its apical width, 1.2–1.4× longer than second tergite. Second tergite 1.2–1.5× longer than third tergite. Length of first to third tergites combined 1.1–1.2× their maximum width. Third tergite weakly evenly curved on posterior margin. Ovipositor sheaths short, weakly thickened, 0.2–0.3× as long as first–third tergites combined.

***Sculpture***. Head densely and small areolate-punctate, vertex sometimes with fine transverse striae posteriorly, frons punctate-shagreened; face densely punctate-reticulate and upper with transverse striation upper, clypeus weakly and sparsely punctate, shagreened between punctures. Mesoscutum and scutellum densely and distinctly punctate. Mesopleuron anteriorly and below sparse punctate and finely shagreened, almost smooth in posterior upper half; metapleuron transversely striate with sparse punctation anteriorly, rugose-reticulate posteriorly. Propodeum submedially with coarse curved transverse keel; often areas distinctly delineated by carinae, with narrow and inversely-pentagonal areola, but sometimes anterior carinae of areola indistinct and areola indistinctly delineated; anterolateral areas elongated, petiolate area short; propodeum usually entirely densely rugulose in basal half, but sometimes almost smooth in posterior half. First and second tergites entirely and third tergite in basal 0.5–0.7 distinctly and densely striate with dense rugosity or densely areolate-rugose; posterior half of third and following tergites densely and finely shagreened to smooth.

***Colour***. Body mainly black, only sometimes head partly dark reddish brown. Antenna black, few basal segments dark reddish brown. Palpi yellow, usually infuscate basally. Fore and middle legs yellowish brown or reddish brown, partly paler; hind leg reddish brown to dark reddish brown, partly black. Fore wing faintly infuscate, without dark band under pterostigma or only with wide darkening. Pterostigma and parastigma dark brown, most veins brown, metacarp (1-R1) pale brown.

**Male**. Body length 1.9–2.3 mm; fore wing length 1.8–2.0 mm. Antenna 20-segmented, evenly setiform, distinctly longer than body. First flagellar segment 2.2–2.4× longer than its apical width, 1.1–1.2× longer than second segment. Penultimate segment ~2.0× longer than its width. Discoidal (discal) cell of fore wing distinctly petiolate. First tergite only weakly longer than second tergite. Second tergite 1.3–1.7× longer than third tergite. Length of first to third tergites combined 1.1–1.3× their maximum width. Head often dark reddish brown to black, sometimes face reddish brown. Hind femur mainly (dark) reddish brown or pale brown. Fore wing hyaline or subhyaline. Pterostigma and parastigma brown, most veins hyaline and transparent. Otherwise similar to female.

##### Distribution.

Russia (Primorskiy Territory, Sakhalin Province: Kuril Islands), Korean Peninsula.

#### ﻿ Paradelius (Sculptomyriola) ghilarovi

Taxon classificationAnimaliaHymenopteraBraconidae

(Belokobylskij, 1988) comb. nov.

3EA94CD3-15D3-5F36-8D70-A049F2F4EBB1

Figs 5 , 8A

Sculptomyriola ghilarovi Belokobylskij, 1988: 147; 1998: 556; Ku et al. 2001: 15; Yu et al. 2016.

##### Material examined.

Russia. Primorskiy Territory: “Primorskiy Territory, 20 km SE of Ussuriysk, Gornotayozhnoe, at light, Budris [col.], 3.IX.1983”, “Holotypus *Sculptomyriolaghilarovi* Belokobylskij”, 1 female (HT) (ZISP); 20 km E of Ussuriysk, GTS, at light, 3, 5 & 6.IX.1983 (E. Budris), 5 females (PTs); 20 km E of Ussuriysk, GTS, at light, 27.VIII.1984 (S. Sinev), 2 females (PTs) (All in ZISP).

South Korea. Gyeongsangbuk-do, Mt. Baekam, Gujuryeong, at light, 10–11.VIII.1999 (D. Ku), 1 female (SMNE).

##### Description.

**Female**. Body length 2.1–2.5 mm; fore wing length 1.9–2.0 mm.

***Head***. Head 2.0–2.3× wider than its medial length (dorsal view), 0.9–1.0× as wide as mesoscutum. Occiput distinctly evenly concave. Head behind eyes distinctly roundly narrowed; transverse diameter of eye 1.5–1.8× larger than length of temple (dorsal view). Ocelli arranged in triangle with base 1.3–1.5× its sides. POL 2.3–2.6× Od, 0.9–1.1× OOL. Eye 1.5–1.6× as high as broad. Malar space 0.8–1.1× basal width of mandible, 0.2–0.3× height of eye. Face convex, its width 1.5–1.7× median height, almost equal to height of eye. Tentorial pits small, distance between pits almost equal to distance from pit to eye. Clypeus high and weakly convex, its width ~ 2.0× median height, 0.6–0.7× width of face; its ventral margin straight medially. Head strongly roundly narrowed below eyes (front view).

**Figure 5.**
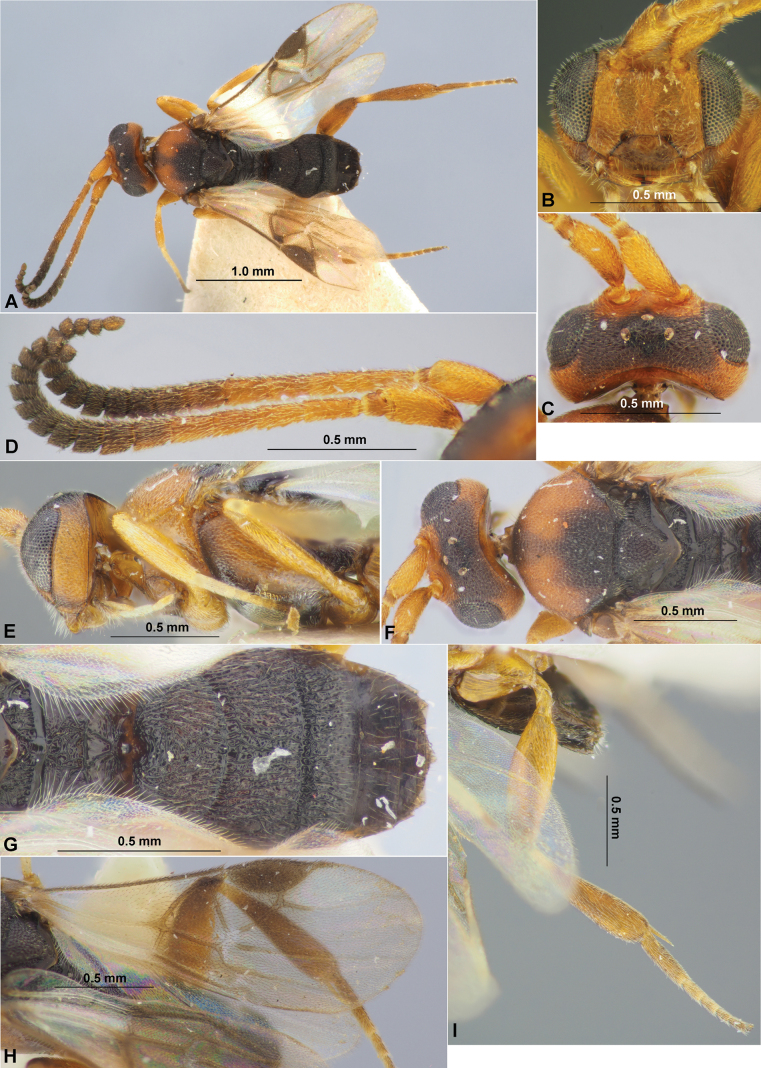
Paradelius (Sculptomyriola) ghilarovi (Belokobylskij, 1988), comb. nov. (female, holotype) **A** habitus, dorsal view **B** head, front view **C** head, dorsal view **D** antenna **E** head and mesosoma, lateral view **F** head and mesosoma, dorsal view **G** propodeum and metasoma, dorsal view **H** wings **I** hind leg.

***Antenna***. Antenna 20-segmented, thickened, setiform, apical segments started from ninth transverse and wide in beginning and subsquare or almost rounded subapically (three–four segments). Scape 2.0–2.3× longer than wide. First flagellar segment 2.4–2.7× longer than its apical width, 1.2–1.4× longer than second segment. Tenth segment 0.6× as long as maximum width. Penultimate segment subround, as long as its width, 0.6–0.7× as long as obtuse apical segment.

***Mesosoma***. Mesosoma 1.5–1.6× longer than maximum height. Mesoscutum highly and curvedly elevated above pronotum (lateral view), 1.3–1.4× as wide as its medial length (dorsal view). Prescutellar depression (scutellar sulcus) shallow, with numerous distinct carinae. Scutellum 0.9× as long as anterior width. Prepectal carina almost entirely absent, sometimes very weakly visible laterally. Precoxal sulcus distinct, oblique, extending below almost throughout all lower part of mesopleuron, rugulose-crenulate.

***Wings***. Fore wing 2.4–2.5× longer than maximum width. Pterostigma 2.5–2.7× longer than its maximum width. Radial vein (r) arising from distal 0.4 of pterostigma; first radiomedial vein (2-SR) arising from radial vein (r) weakly distant from pterostigma. Present short first (r) and long second (3-SR) abscissae of radial vein, second abscissa (3-SR) curved anteriorly and almost straight posteriorly, sclerotised in basal 0.2–0.3 and desclerotised on remaining part, reaching as track distal margin of wing. Radial (marginal) cell weakly shortened, 2.7–2.8× longer than its maximum width. Sclerotised basal part of metacarp (1-R1) short, ~ 0.2× as long as pterostigma. First radiomedial vein (2-SR) mainly distinctly sclerotised and pigmented, 5.0–7.0× longer than recurrent vein (m-cu). Recurrent vein (m-cu) weakly postfurcal to first radiomedial vein (2-SR), posteriorly weakly convergent distally with basal vein (1-M). Discoidal (discal) cell broadly sessile anteriorly, ~ 1.3× longer than its maximum width. Nervulus (cu-a) long, subperpendicular to longitudinal anal vein (1-1A), weakly postfurcal, distance between basal vein (1-M) and nervulus (cu-a) 0.2–0.3× nervulus (cu-a) length. Hind wing 3.0–3.6× longer than maximum width. First abscissa of mediocubital vein (M+CU) ~ 2.5× longer than second abscissa (1-M).

***Legs***. Hind coxa short and high, 1.4–1.5× longer than maximum width, 1.5–1.7× longer than propodeum (lateral view). Hind femur 2.7–3.1× longer than its maximum width. Hind tibia distinctly claviform, ~ 4.5× longer than maximum width, ~ 0.7× as wide as hind femur; longest inner tibial spur ~ 0.5× hind basitarsus length. Hind tarsus 0.9–1.0× as long as hind tibia, its basitarsus 0.8–0.9× as long as second–fifth segments combined, 2.6–2.8× longer than second segment, 3.0–3.3× longer than fifth segments (without pretarsus).

***Metasoma***. Metasoma 0.8–0.9× as long as mesosoma. All tergites (especially first to third ones) distinctly sclerotised; first and second sutures distinct, narrow and crenulate, first suture strongly curved, second one weakly curved. Medial length of first tergite 0.6–0.7× its apical width, 1.3–1.4× longer than second tergite. Second tergite ~ 1.3× longer than third tergite. Length of first to third tergites combined 1.1–1.2× their maximum width. Third tergite almost straight on posterior margin. Ovipositor sheaths short, weakly thickened, 0.2–0.3× as long as first–third tergites combined.

***Sculpture***. Head densely and small areolate-punctate with additional small granulation, frons and face densely transversely curvedly striae with dense reticulation between striae, clypeus distinctly densely punctate and smooth between punctures. Mesoscutum and scutellum densely and distinctly punctate-areolate. Mesopleuron entirely or widely and rather sparsely punctate, smooth between punctulae, sometimes entirely smooth in posterior upper half; metapleuron entirely rugose-areolate. Propodeum medially with coarse transverse curved keel; areas rather distinctly or relatively finely delineated by carinae, with rather wide and inversely-pentagonal areola, anterolateral areas rela­tively wide, petiolate area short and trapezoid; propodeum entirely or almost entirely densely rugulose-reticulate. First and second tergites entirely and third tergite in basal 0.8–0.9 distinctly and densely curvedly striate with rugosity; following tergites densely and very finely shagreened to smooth.

***Colour***. Head mainly pale reddish brown, darkened only dorsally on vertex. Mesosoma dorsally mainly black, but pale reddish brown in anterior one–fifth and on large medial area of mesopleuron or almost entirely laterally. Metasoma entirely black. Antenna brownish yellow to pale reddish brown in basal 0.5–0.7, dark brown or black in apical 0.3–0.5. Palpi pale yellow, but distinctly infuscate basally. Legs mainly pale reddish brown to partly reddish brown, sometimes hind tibia and always hind tarsus infuscate; all tibia yellow or pale yellow basally. Fore wing partly faintly infuscate, hyaline in basal one–third, with rather distinct and wide dark sport medially (under pterostigma and along basal (1-M) vein). Pterostigma and parastigma dark brown; most veins brown, but veins in basal one third and apically pale, hyaline.

**Male**. Unknown.

##### Distribution.

Russia (Primorskiy Territory), Korean Peninsula.

#### ﻿ Paradelius (Sculptomyriola) koreanus

Taxon classificationAnimaliaHymenopteraBraconidae

Belokobylskij & Ku sp. nov.

F1003A82-8356-554A-A219-C971C08F1509

https://zoobank.org/A6D53983-6522-47C5-8B7F-4DA041E9B399

Figs 6 , 8B

##### Type material.

***Holotype***, female, South Korea, Gyeongsangnam-do, Jinju-si, Gajwa-dong, Light trap, 2–3.VIII.2000 (Tae-Ho An) (NIBR).

##### Description.

**Female**. Body length 1.5 mm; fore wing length 1.4 mm.

***Head***. Head almost twice wider than its medial length (dorsal view), 1.1× wider than mesoscutum. Occiput distinctly evenly concave. Head behind eyes weakly convex in anterior half and roundly narrowed in posterior half; transverse diameter of eye twice larger than length of temple. Ocelli arranged in triangle with base 1.25× its sides. POL 1.5× Od, 0.8× OOL. Eye 1.5× as high as broad. Malar space 0.6× basal width of mandible, 0.15× height of eye. Face convex, width of face 1.3× its median height, almost equal to height of eye. Tentorial pits small, distance between pits 1.3× distance from pit to eye. Clypeal suture distinct and narrow. Clypeus wide and weakly convex, its width 2.4× median height, 0.7× width of face; almost straight on lower margin medially. Head distinctly roundly narrowed below eyes (front view). Occipital carina dorsally complete, but weak.

**Figure 6.**
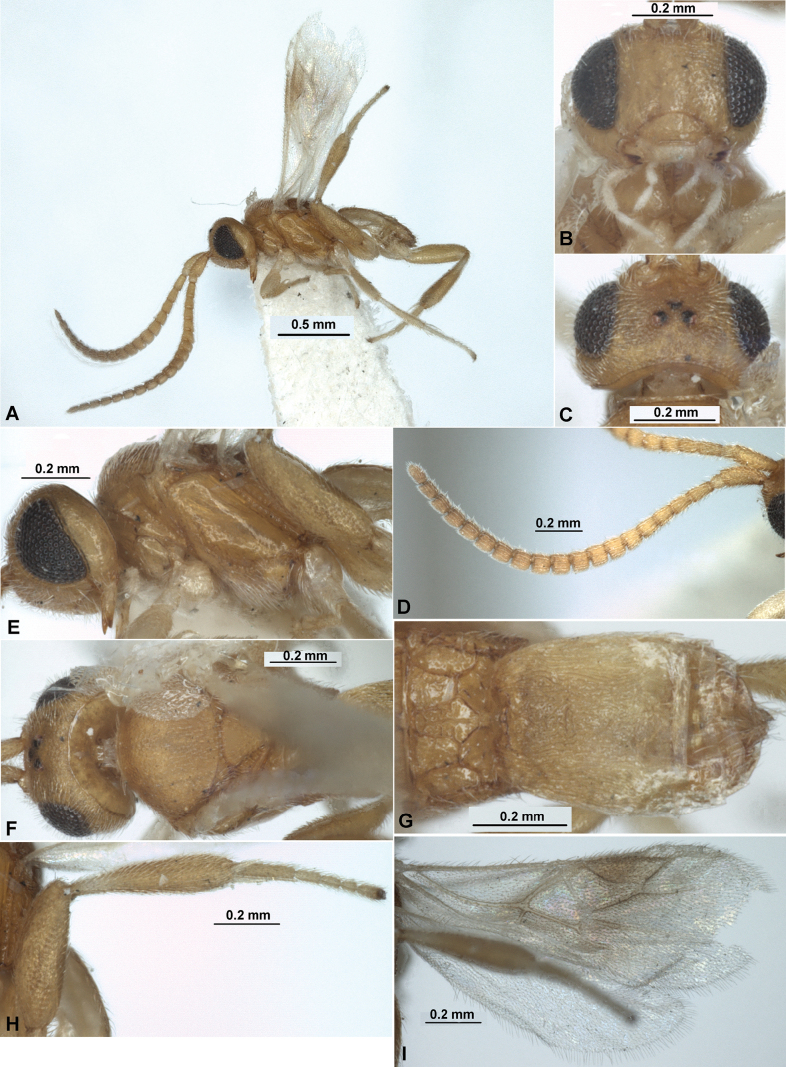
Paradelius (Sculptomyriola) koreanus Belokobylskij & Ku, sp. nov. (female, holotype) **A** habitus, lateral view **B** head, front view **C** head, dorsal view **D** antenna **E** head and mesosoma, lateral view **F** head and mesosoma, dorsal view **G** propodeum and metasoma, dorsal view **H** hind leg **I** wings.

***Antenna***. Antenna 20-segmented, 1.3× longer than body, rather thick, submedial segments short and wide. Scape 2.0× longer than wide. First flagellar segment almost twice longer than its apical width, 1.3× longer than second segment; seven to 15^th^ segments subsquare or weakly transverse, 1.0–1.2× as wide as their length. Penultimate segment 1.2× longer than its width, 0.7× as long as apical segment.

***Mesosoma***. Mesosoma 1.5× longer than maximum height. Mesoscutum highly and roundly elevated above pronotum (lateral view), 1.3× as wide as medial length (dorsal view). Prescutellar depression (scutellar sulcus) very narrow and shallow, with distinct numerous carinae. Scutellum 0.8× as long as its anterior width. Prepectal carina weakly present laterally and widely absent ventrally. Precoxal sulcus distinctly impressed, long and curved, extending below almost throughout mesopleuron, crenulate.

***Wings***. Fore wing 2.8× longer than maximum width. Pterostigma 3.4× longer than its maximum width. Radial vein (r) arising from distal 0.3 of pterostigma. First radial vein (r) present, short, subvertical, 0.3× as long as width of pterostigma. Second radial vein (3-SR) sclerotised in basal half and unsclerotised in apical half, reaching as track distal margin of wing. Radial (marginal) cell distinctly shortened, almost 3.0× longer than its maximum width. Metacarp (1-R1) unsclerotised distally, its sclerotised basal part ~ 0.5× as long as pterostigma. First radiomedial vein (2-SR) distinctly sclerotised, 7.2× longer that first radial abscissa (r), 4.5× longer than recurrent vein (m-cu). Recurrent vein (m-cu) postfurcal to first radiomedial vein (2-SR), posteriorly subparallel with basal vein (1-M) and 0.25× as long as basal vein (1-M). Discoidal (discal) cell narrowly sessile anteriorly, 1.4× longer than its maximum width. Nervulus (cu-a) weakly postfurcal, subperpendicular and oblique to longitudinal anal vein (1-1A), distance between basal vein (1-M) and nervulus (cu-a) 0.2× nervulus (cu-a) length. Hind wing 3.2× longer than maximum width. First abscissa of mediocubital vein (M+CU) 2.0× longer than second abscissa (1-M).

***Legs***. Hind coxa long and rather low, 1.8× longer than maximum width, 1.6× longer than propodeum (lateral view). Hind femur thickened and short, 3.0× longer than maximum width. Hind tibia distinctly claviform, strongly thickened apically, 4.2× longer than maximum width, 0.8× as wide as hind femur; its longest inner spur 0.7× hind basitarsus length. Hind tarsus 0.9× as long as hind tibia. Basitarsus of hind leg 0.6× as long as second–fifth segments combined, 2.5× longer than second segment, ~3.0× longer than fifth segments (without pretarsus).

***Metasoma***. Metasoma 0.9× as long as mesosoma. All tergites distinctly sclerotised; first and second sutures rather distinct, almost complete, narrow and curved. Medial length of first tergite ~ 0.4× its apical width, almost as long as second tergite. Second tergite 1.6× longer than third tergite. Length of first to third tergites combined 1.2× their maximum width. Third tergite weakly curved on posterior margin. Hypopygium setose, reaching apex of metasoma. Ovipositor sheaths very short not projected behind tip of metasoma, ~ 0.2× as long as first–third tergites combined.

***Sculpture***. Head densely areolate-rugulose with additional dense granulation; face densely punctate, with transverse striation in upper half, clypeus weakly and sparsely punctate. Mesoscutum and scutellum densely foveolate with fine additional granulation. Mesopleuron anteriorly and below finely foveolate-punctate, almost smooth upper and posteriorly; metapleuron rugose-striate, but almost smooth medially. Propodeum entirely distinctly reticulate-carinate, with strong and complete carinae separated long and inversely-pentagonal areola, elongated anterolateral and subround distolateral areas, with short and subsquare petiolate area. First and second tergites entirely distinctly and densely rugose-striate, first tergite medially widely and densely rugose; third tergite mainly smooth, only finely rugulose in narrow basomedial part.

***Colour***. Body entirely brownish yellow, only antenna very faintly infuscate apically. Palpi pale yellow. Legs basally yellow, brownish yellow on remaining part. Fore wing subhyaline basally and apically, with distinctly infuscate and wide band medially. Pterostigma pale brown, yellow in apical one–third.

**Male**. Unknown.

##### Comparative diagnosis.

Paradelius (Sculptomyriola) koreanus sp. nov. distinctly differs from all eastern Palaearctic species of this genus with sessile anteriorly discoidal (discal) cell of fore wing (Belokobylskij, 1988, 1998) by almost completely brownish yellow coloration of the body, only medially sculptured third metasomal tergite and the short and weakly transverse or subsquare medial segments of antenna.

##### Etymology.

This species is named after the Korean Peninsula, where new species was collected.

##### Distribution.

Korean Peninsula.

#### ﻿ Paradelius (Sculptomyriola) sinevi

Taxon classificationAnimaliaHymenopteraBraconidae

(Belokobylskij, 1998) comb. nov.

9B22B398-B848-5FBB-8EEF-9D6715C68923

Figs 7 , 8C

Sculptomyriola sinevi Belokobylskij, 1998: 555; Yu et al. 2016.

##### Material examined.

Russia. Primorskiy Territory: “Primorskiy Territory, 20 km SE of Ussuriysk, at light, 31.VII.1991, Belokobylskij [col.]”, “Holotype *Sculptomyriolasinevi* Belokobylskij”, 1 female (HT) (ZISP); 7 km S of Zanadvorovka, at light, 13.VIII.1984 (S. Sinev), 1 male (PT); ‘Kedrovaya Pad’ Nature Reserve, cordon of Sukhaya Rechka, 6.VIII.1988 (E. Budris), 1 male (PT); ‘Kedrovaya Pad’’ Nature Reserve, at light, 7.VIII.1988 (E. Budris), 1 female (PT); 10 km SE of Partizansk, bush on slopes of hill, 11.VII.1996 (S. Belokobylskij), 1 male (PT); 20 km SE of Ussuriysk, at light, 27.VIII.1984 (S. Sinev), 1 female (PT); 20 km SE of Ussuriysk, Gornotayozhnoe, at light, 26.VII.1999 (S. Sinev), 1 female; 30 km E of Spassk-Dal’niy, forest, 27.VIII.1992 (S. Belokobylskij), 1 female (PT) (All in ZISP).

**Figure 7.**
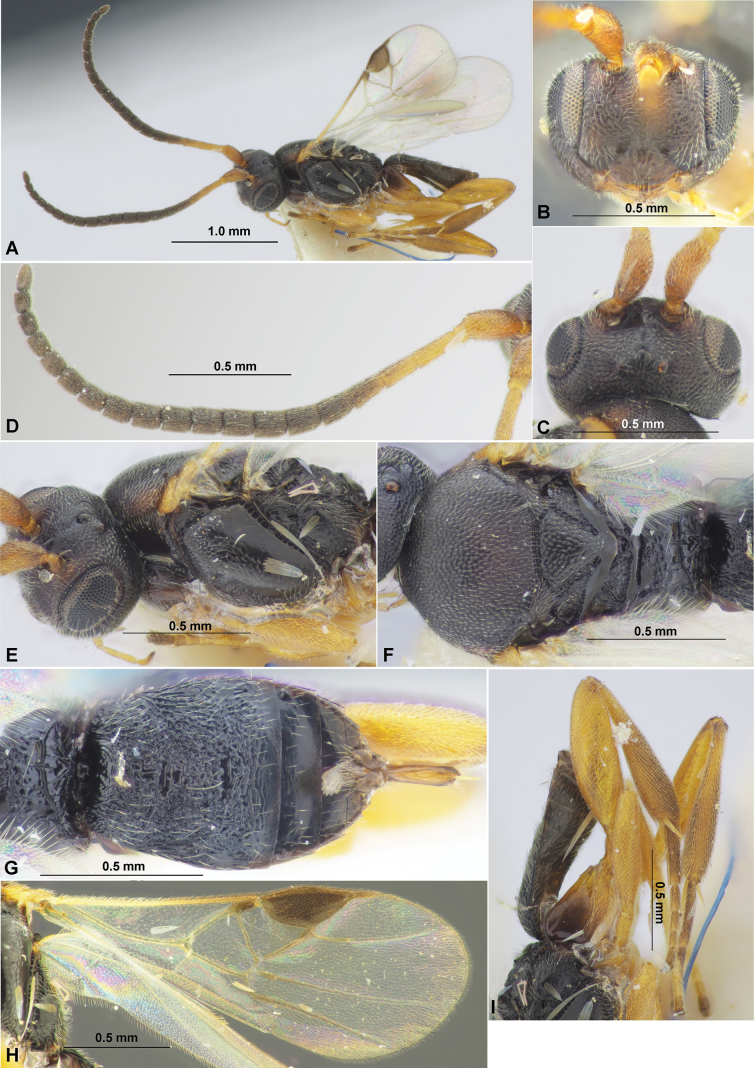
Paradelius (Sculptomyriola) sinevi (Belokobylskij, 1998), comb. nov. (female, holotype) **A** habitus, lateral view **B** head, front view **C** head, dorsal view **D** antenna **E** head and mesosoma, lateral view **F** mesosoma, dorsal view **G** propodeum and metasoma, dorsal view **H** wings **I** hind leg.

##### Description.

**Female**. Body length 2.2–2.4 mm; fore wing length 1.8–1.9 mm.

***Head***. Head 1.7–1.9× wider than its medial length (dorsal view), 0.9–1.0× as wide as mesoscutum. Occiput distinctly evenly concave. Head behind eyes distinctly roundly narrowed; transverse diameter of eye 1.3–1.4× larger than length of temple (dorsal view). Ocelli arranged in triangle with base 1.5–1.6× its sides. POL ~ 2.5× Od, 0.8–1.0× OOL. Eye 1.4–1.6× as high as broad. Malar space 1.1–1.3× basal width of mandible, 0.30–0.35× height of eye. Face weakly convex, width of face ~ 1.5× its median height, 1.1–1.3× height of eye. Tentorial pits small, distance between pits almost equal to distance from pit to eye. Clypeus high and weakly convex, its width ~ 2.0× median height, 0.7× width of face; ventral margin of clypeus weakly curved. Head strongly roundly narrowed below eyes (front view).

***Antenna***. Antenna 20-segmented, thickened medially, weakly narrowed basally and apically, subfiliform, medial segments started from eighth to 17^th^ weakly elongated or sometimes subsquare. Scape 2.0–2.2× longer than wide. First flagellar segment 2.6–2.7× longer than its apical width, ~ 1.3× longer than second segment. Tenth segment 1.20–1.25× longer than its maximum width. Penultimate segment 1.2–1.3× longer than its width, 0.7–0.8× as long as obtuse apical segment.

***Mesosoma***. Mesosoma 1.5–1.6× longer than maximum height. Mesoscutum highly and curvedly elevated above pronotum (lateral view), 1.3–1.5× as wide as its medial length (dorsal view). Prescutellar depression (scutellar sulcus) shallow, with numerous distinct carinae. Scutellum 0.9× as long as anterior width. Prepectal carina widely absent, rarely only weakly visible laterally. Precoxal sulcus distinct, wide, strongly curved, extending below almost throughout all lower part of mesopleuron, rugulose-crenulate.

**Figure 8.**
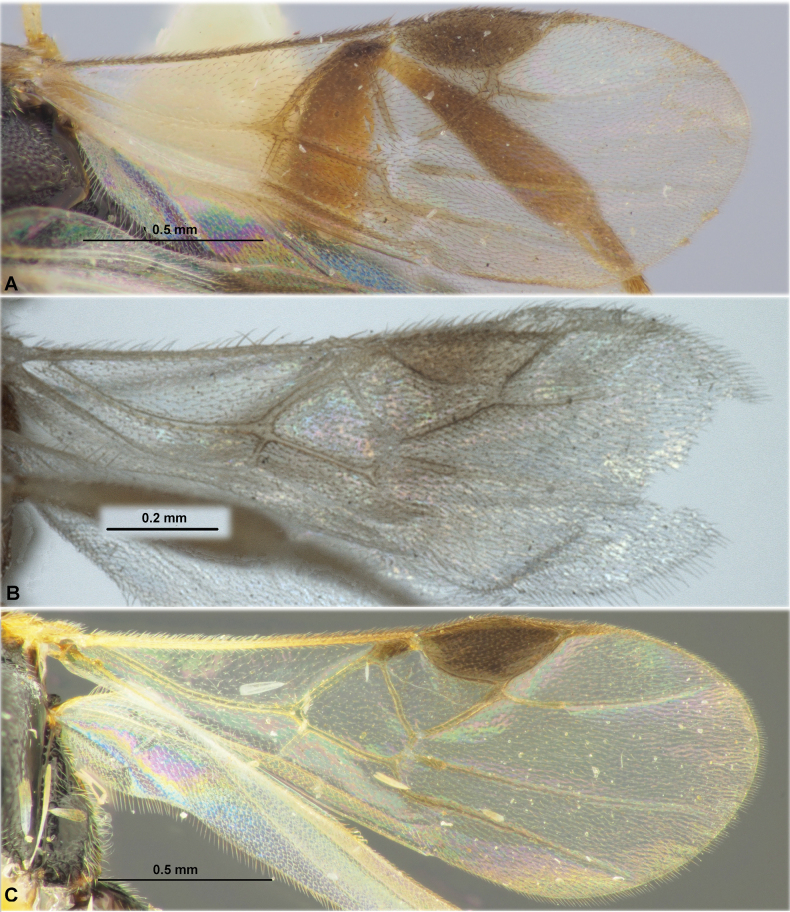
Fore wings. **A**Paradelius (Sculptomyriola) ghilarovi (Belokobylskij) **B**P. (Sc.) koreanus sp. nov. **C**P. (Sc.) sinevi (Belokobylskij).

***Wings***. Fore wing 2.3–2.6× longer than maximum width. Pterostigma 2.3–2.5× longer than its maximum width. Radial vein (r) arising from distal 0.40–0.45 of pterostigma, radiomedial vein (2-SR) arising from radial vein (r) weakly separated from pterostigma. Present short first (r) and second (3-SR) abscissa of radial vein, second abscissa (3-SR) entirely evenly curved, weakly sclerotised in basal 0.25 and desclerotised on remaining part, reaching as track distal margin of wing. Radial (marginal) cell shortened, ~ 3.0× longer than its maximum width. Metacarp (1-R1) short, pigmented, its sclerotised basal part 0.3× as long as pterostigma. First radiomedial vein (2-SR) mainly distinctly sclerotised and pigmented, 6.5–7.5× longer than short recurrent vein (m-cu). Recurrent vein (m-cu) distinctly postfurcal to first radiomedial vein (2-SR), approximately as long as second medial abscissa (2-SR+M), posteriorly subparallel with basal vein (1-M). Discoidal (discal) cell narrowly sessile anteriorly, 1.3–1.4× longer than its maximum width. Nervulus (cu-a) oblique to longitudinal anal vein (1-1A), postfurcal, distance between basal vein (1-M) and nervulus (cu-a) ~ 0.4× nervulus (cu-a) length. Hind wing 3.3–3.5× longer than maximum width. First abscissa of mediocubital vein (M+CU) 2.0–2.5× longer than second abscissa (1-M).

***Legs***. Hind coxa long and high, 1.5–1.6× longer than maximum width, 1.1–1.2× longer than propodeum (lateral view). Hind femur 3.0–3.4× longer than maximum width. Hind tibia distinctly claviform, 4.2–4.4× longer than maximum width, 0.8× as wide as hind femur; longest inner tibial spur 0.6× hind basitarsus length. Hind tarsus 0.9× as long as hind tibia, its basitarsus 0.8× as long as second–fifth segments combined, 2.3–2.7× longer than second segment, 3.0–3.7× longer than fifth segments (without pretarsus).

***Metasoma***. Metasoma 0.8–0.9× as long as mesosoma. All tergites (especially first to third ones) distinctly sclerotised; first and second sutures distinct, but narrow, first suture strongly curved, second one weakly curved. Medial length of first tergite 0.5× its apical width, 1.0–1.2× as long as second tergite. Second tergite 1.1–1.4× longer than third tergite. Length of first to third tergites combined 1.0–1.1× their maximum width. Third tergite weakly evenly curved on posterior margin. Ovipositor sheaths weakly thickened, short, 0.3–0.4× as long as first–third tergites combined.

***Sculpture***. Head densely and small areolate-punctate, partly arranged in transverse curved lines, frons densely reticulate in upper half and striate in lower half; face densely curvedly transverse striate and with dense additional punctation, clypeus with sparse punctation, smooth between punctures. Mesoscutum and scutellum very densely and distinctly punctate, sometimes partly with small areolae. Mesopleuron mainly smooth in posterior upper half and in narrow area upper precoxal sulcus, distinctly and rather sparse punctate with reticulation in anterior lower half; metapleuron rugose-areolate with striation, usually with two small, subround and almost smooth areas. Propodeum submedially with coarse transverse and curved keel; areas not clearly delineated by carinae, areola and anterolateral areas absent; propodeum almost entirely densely rugulose-reticulate. First and second tergites entirely and third tergi­te in basal 0.7–0.8 (at least laterally) distinctly and densely rugose-reticulate, sometimes third tergite medially in basal 0.7 with transverse curved striae; apical part of third tergite and following tergites smooth.

***Colour***. Body black, rarely head dark reddish brown. Antenna yellow or brownish yellow in basal 0.3, black in apical 0.7, scape pale reddish brown. Palpi pale brown or yellow. Legs pale reddish brown, fore and middle legs paler, hind coxa in basal half, hind tibia mostly or widely and hind tarsus almost entirely reddish brown to dark reddish brown; tibial spurs yellow. Fore wing hyaline or very faintly infuscate, without dark bands. Pterostigma and parastigma dark brown, pterostigma sometimes faintly paler basally and apically; most veins pale brown or yellow.

**Male**. Body length 2.3–2.5 mm; fore wing length 1.9–2.0 mm. Antenna 20-segmented, less thickened, evenly setiform, longer than body, its basal one–third or half pale reddish brown to dark reddish brown. First flagellar segment 2.2–2.3× longer than its apical width, 1.10–1.15× longer than second segment; tenth segment 1.5–2.0× longer than its maximum width; penultimate segment 2.2–2.3× longer than its width. Mesopleuron widely smooth or sparsely to very sparsely punctate. First tergite 1.2–1.5× as long as second tergite. Third tergite rugulose-reticulate only in basal 0.2–0.3; following tergites usually weakly shagreened. Hind leg mainly reddish brown to dark reddish brown, almost black partly. Fore wing entirely hyaline; most veins subhyaline or pale. Otherwise similar to female.

##### Distribution.

Russia (Primorskiy Territory).

#### ﻿ Subgenus Sinadelius

Taxon classificationAnimaliaHymenopteraBraconidae

He & Chen, 2000

748D1CD2-F1FE-50B9-9AAF-1DE216E5F102

**Figure 9.**
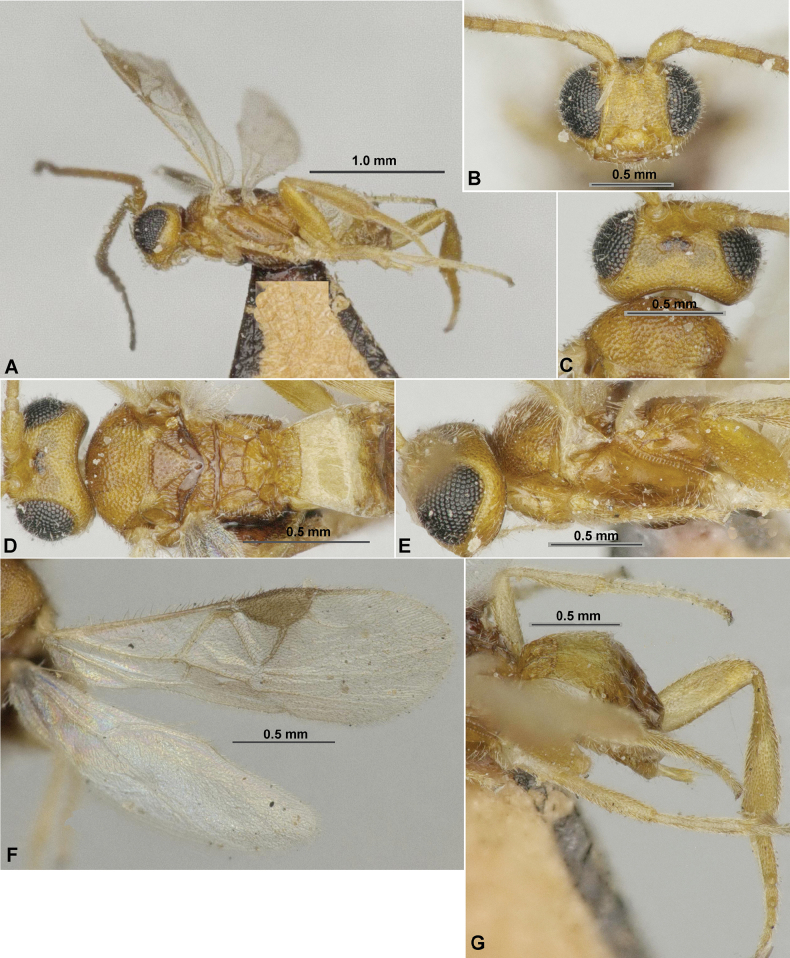
Paradelius (Sinadelius) guangxiensis (He & Chen, 2000), comb. nov. (female, holotype) **A** habitus, lateral view **B** head and base of antenna, front view **C** head, dorsal view **D** head, mesosoma and base of metasoma, dorsal view **E** head and mesosoma, lateral view **F** wings **G** metasoma and hind leg, lateral view.

**Figure 10.**
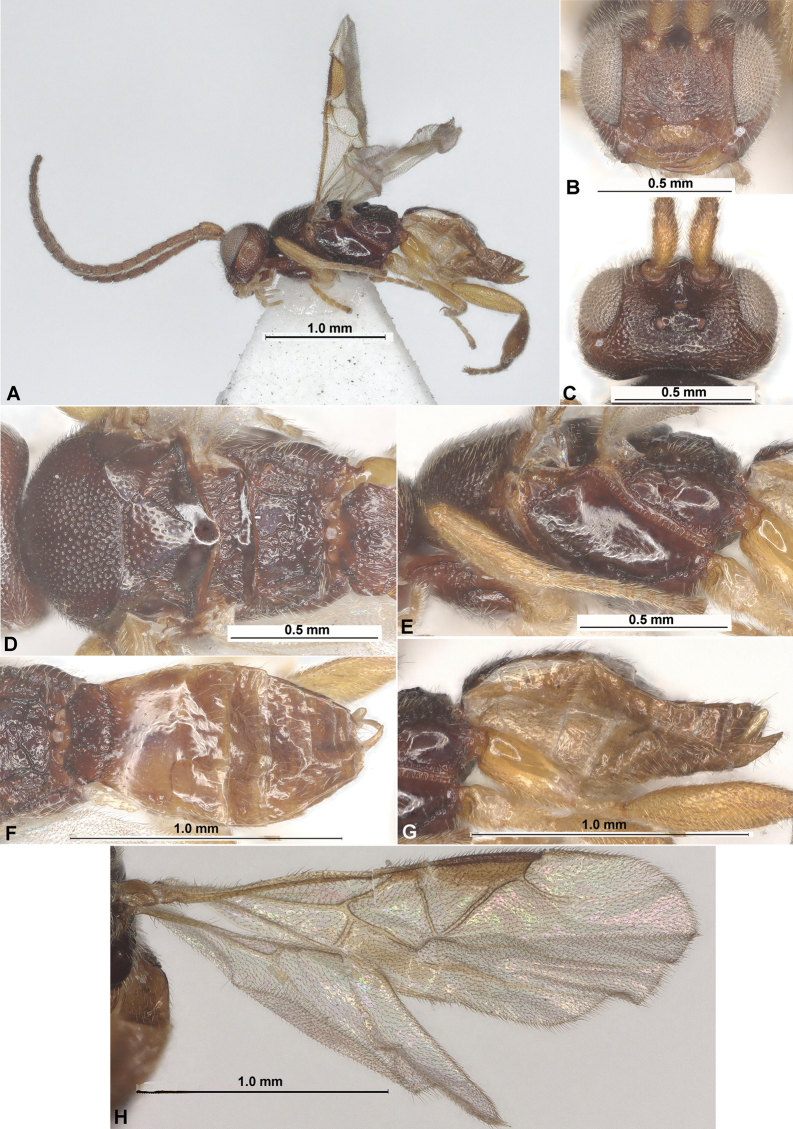
Paradelius (Sinadelius) nigricans (He & Chen, 2000), comb. nov. (male, holotype) **A** habitus, lateral view **B** head, front view **C** head, dorsal view **D** mesosoma, dorsal view **E** mesosoma, lateral view **F** propodeum and metasoma, dorsal view **G** propodeum and metasoma, lateral view **H** wings.

**Figure 11.**
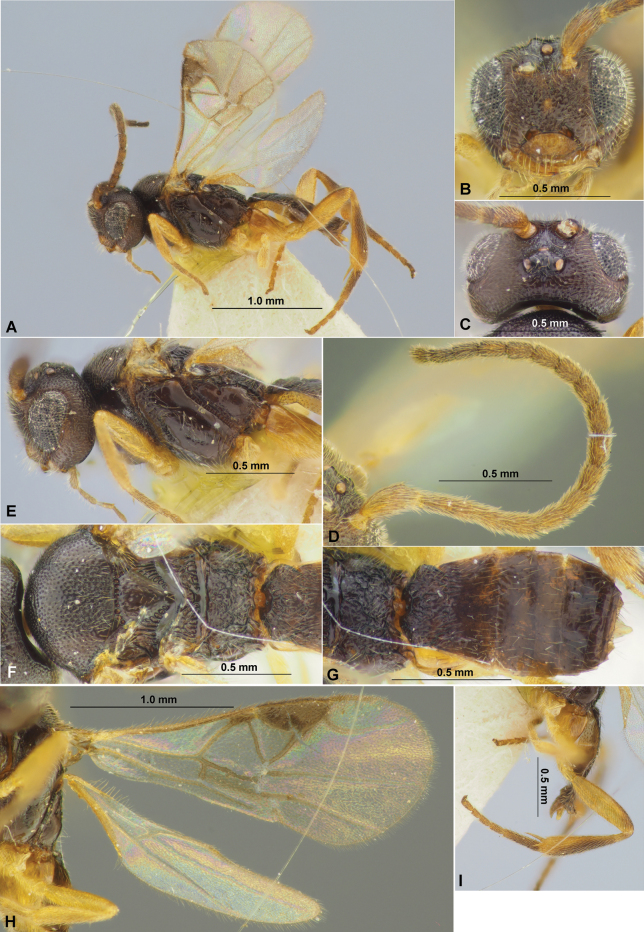
Paradelius (Sinadelius) ussuriensis Belokobylskij, 1988 (male, holotype) **A** habitus, lateral view **B** head, front view **C** head, dorsal view **D** antenna **E** head and mesosoma, lateral view **F** mesosoma and base of metasoma, dorsal view **G** propodeum and metasoma, dorsal view **H** wings **I** hind leg.

**Figure 12.**
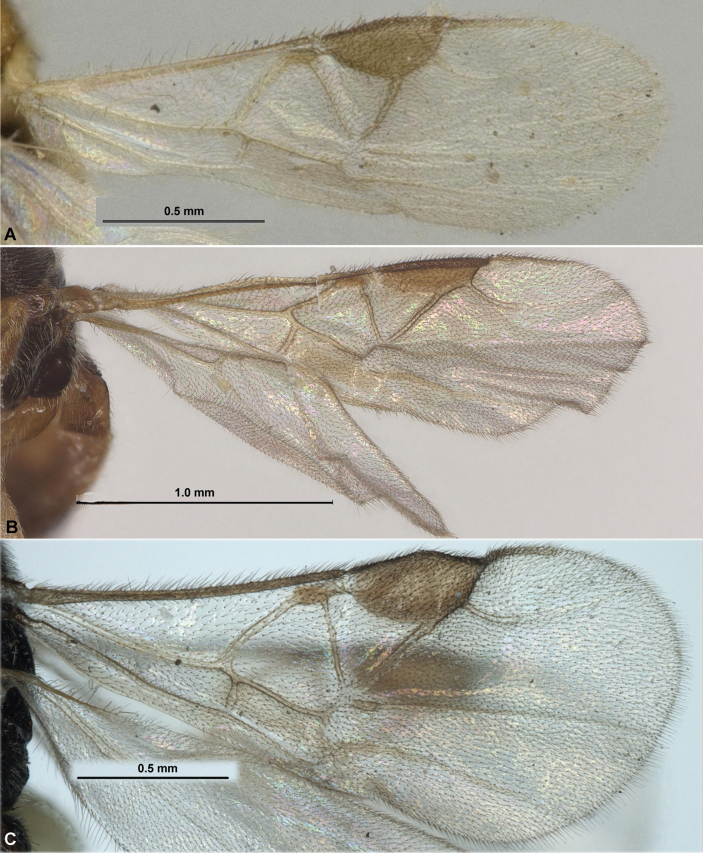
Fore wings. **A**Paradelius (Sinadelius) guangxiensis (He & Chen) **B**P. (S.) nigricans (He & Chen) **C**P. (S.) ussuriensis Belokobylskij.

Figs 9 , 10 , 11 , 12

##### Description of subgenus.

Vertex densely reticulate-punctate. Ocelli arranged in obtuse triangle. Eye covered by dense and long setae. Antenna long, thickened (especially medially) and setiform. Flagellar segments always longitudinal in apical half of antenna. Mesoscutum densely and distinctly punctate. Prescutellar depression short and crenulate. Prepectal carina laterally absent. Precoxal sulcus distinct, relatively wide, entirely crenulate or rugulose. Propodeum almost entirely rugulose-reticulate, often with areas delineated by carinae. Fore wing with large pterostigma. Radial vein (r) always with one abscissa, arising from pterostigma distinctly distant from first radiomedial vein (2-SR). Discoidal (discal) cell anteriorly always sessile. Recurrent vein (m-cu) weakly postfurcal or interstitial to first radiomedial vein (2-SR). Hind femur wide; hind tibia distinctly clavate. First tergite of metasoma subparallel or weakly narrowed towards apex behind distinct spiracular tubercles, entirely rugose-reticulate. Suture between first and second tergites present, usually distinct, densely crenulate. Second and following tergites smooth, only second one shortly rugulose basally; suture between second and third tergites very weak, shallow and smooth (as trace). Ovipositor short.

#### ﻿ Paradelius (Sinadelius) guangxiensis

Taxon classificationAnimaliaHymenopteraBraconidae

(He & Chen, 2000) comb. nov.

FC09D618-26F2-564C-87DD-2AA28CF219C3

Figs 9 , 12A

Sinadelius guangxiensis He & Chen in He et al. 2000: 682; Yu et al. 2016.

##### Material examined.

China: Guangxi, Longzhou, Nonggang, 20.V. 1985 (He Junhua col.), No. 824419, 1 female (HT) (ZJUH); same label as in holotype, 1 female (PT) (ZJUH).

##### Description.

**Female**. Body length 1.8 mm; fore wing length 1.8 mm.

***Head***. Head 1.9× wider than its medial length (dorsal view), 1.2× wider than mesoscutum. Occiput weakly concave. Head behind eyes distinctly evenly roundly narrowed; transverse diameter of eye ~ 2.5× larger than length of temple (dorsal view). Ocelli arranged in triangle with base 1.5× its sides. POL ~ 2.0× Od, 0.9× OOL. Eye large, 1.5× as high as broad. Malar space ~0.5× basal width of mandible, 0.1× height of eye. Face weakly convex, width of face 1.2× its median height, 0.9× height of eye. Tentorial pits distinct, distance between pits 1.3× distance from pit to eye. Clypeus rather high and convex, its width 2.2× median height, 0.8× width of face; its ventral margin almost straight medially. Head distinctly roundly narrowed below eyes (front view).

***Antenna***. Antenna 22-segmented, weakly thickened, weakly setiform, with elongated medial segments. Scape ~ 2.0× longer than wide. First flagellar segment 3.3× longer than its apical width, 1.8× longer than second segment.

***Mesosoma***. Mesosoma 1.7× longer than maximum height. Mesoscutum highly and convex-curvedly elevated above pronotum (lateral view), ~ 2.0× as wide as its medial length (dorsal view). Prescutellar depression (scutellar sulcus) shallow, straight, with distinct numerous carinae. Scutellum almost as long as anterior width. Prepectal carina not visible laterally. Precoxal sulcus distinct, narrow, short, oblique, crenulate.

***Wings***. Fore wing ~ 3.0× longer than maximum width. Pterostigma ~ 2.0× longer than its maximum width. Radial vein (r) arising from distal 0.2 of pterostigma; radiomedial vein (2-SR) arising almost from middle of pterostigma and strongly separated from radial vein (r). Present only single and evenly curved abscissa of radial vein (r), which finely sclerotised in basal 0.25 and desclerotised on remaining part, reaching as track distal margin of wing. Radial (marginal) cell weakly shortened, 3.0× longer than its maximum width. Sclerotised part of metacarp (1-R1) relatively long, faintly pigmented, 0.6× as long as pterostigma. First radiomedial vein (2-SR) weakly curved, sclerotised and brown, ~ 4.3× longer than recurrent vein (m-cu). Recurrent vein (m-cu) weakly postfurcal to first radiomedial vein (2-SR), posteriorly subparallel posteriorly with basal vein (1-M). Discoidal (discal) cell broadly sessile anteriorly, 1.4× longer than its maximum width. Nervulus (cu-a) perpendicular to longitudinal anal vein (1-1A), postfurcal, distance between basal vein (1-M) and nervulus (cu-a) 0.6× nervulus (cu-a) length. Hind wing ~ 3.6× longer than maximum width. First abscissa of mediocubital vein (M+CU) 1.8× longer than second abscissa (1-M).

***Legs***. Hind femur ~ 3.5× longer than maximum width. Hind tibia claviform, distinctly widened distally. 4.8× longer than maximum width, 0.8× as wide as hind femur. Hind tarsus ~ 0.9× as long as hind tibia, its basitarsus 0.8× as long as second–fifth segments combined. Second segment of hind tarsus 0.35× as long as basitarsus.

***Metasoma***. Metasoma approximately as long as mesosoma. All tergites rather distinctly sclerotised. First tergite with weak and thick spiracular tubercles, strongly widened from base to spiracular tubercles situated submedially on tergi­te, then almost subparallel-sided towards posterior margin of tergite. First suture distinct, rather deep, weakly curved, densely crenulate. Medial length of first tergi­te 0.5× its maximum width at level of spiracles, 0.6× as long as second tergite. Second suture present, but very fine and almost straight. Second tergite 1.4× longer than third tergite. Length of first to third tergites combined 0.9× their maximum width. Third tergite almost straight on posterior margin. Ovipositor sheaths weakly thickened, short, 0.4× as long as first–third tergites combined.

***Sculpture***. Vertex densely and small punctate-reticulate; face distinctly transverse striate, with reticulation between striae, laterally below partly almost smooth; clypeus finely rugulose to smooth. Mesoscutum entirely densely punctate, smooth between punctulae, without very dense punctation in its medioposterior area. Scutellum widely smooth and with small sparse punctulae. Mesopleuron almost entirely smooth. Propodeum with distinct and strongly curved submedial transverse keel, without areola, with wide and narrow petiolate area posteriorly; anterolateral areas distinctly delineated by carinae, almost smooth and with punctation, weakly reticulate marginally; propodeum mainly sparsely and rather finely rugose-reticulate. First tergite entirely distinctly rugose-reticulate. Second tergite entirely smooth. Remaining tergites smooth.

***Colour***. Body pale reddish brown or yellowish brown; second and basal one–third of third tergites pale yellow. Antenna mainly pale reddish brown, infuscate towards apex. Palpi yellow. Legs mainly yellow or pale brown, hind tibia and tarsus faintly darkened. Wing mainly subhyaline, with infuscation below pterostigma. Pterostigma and parastigma brown, most veins pale brown, first radiomedial vein (2-SR) brown.

**Male**. Unknown.

##### Distribution.

China (Guangxi Autonomous Region).

#### ﻿ Paradelius (Sinadelius) nigricans

Taxon classificationAnimaliaHymenopteraBraconidae

(He & Chen, 2000) comb. nov.

F5E9D0C6-0234-555B-98A6-0B9F6BA81014

Figs 10 , 12B

Sinadelius nigricans He & Chen in He et al. 2000: 682; Yu et al. 2016.

##### Material examined.

China: Liaoning, Shenyang, Dongling, 21.VI.1994 (Lou Juxian col.), No. 947731, 1 male (HT) (ZJUH).

##### Description.

**Male**. Body length 2.4 mm; fore wing length 2.1 mm.

***Head***. Head 1.7× wider than its medial length (dorsal view), 1.1× wider than mesoscutum. Occiput weakly concave. Head behind eyes evenly roundly narrowed; transverse diameter of eye 1.5× larger than length of temple (dorsal view). Ocelli arranged in triangle with base 1.2× its sides. POL 2.5× Od, 0.8× OOL. Eye 1.3× as high as broad. Malar space ~ 0.7× basal width of mandible, 0.2× height of eye. Face weakly convex, width of face 1.5× its median height, ~ 1.1× height of eye. Tentorial pits distinct, distance between pits 1.4× distance from pit to eye. Clypeus rather low and distinctly convex, its width 2.3× median height, 0.7× width of face; its ventral margin almost straight. Head distinctly roundly narrowed below eyes (front view).

***Antenna***. Antenna 22-segmented, weakly thickened, weakly setiform, with medial segments elongated. Scape ~ 2.0× longer than wide. First flagellar segment 3.3× longer than its apical width, 1.5× longer than second segment. Tenth segment ~ 2.0× longer than its maximum width. Subapical segment 1.6× longer than its width.

***Mesosoma***. Mesosoma 1.7× longer than maximum height. Mesoscutum highly and convex-curvedly elevated above pronotum (lateral view), 2.0× as wide as its medial length (dorsal view). Prescutellar depression (scutellar sulcus) shallow, straight, with distinct numerous carinae. Scutellum 1.1× as long as anterior width. Prepectal carina almost not visible laterally. Precoxal sulcus distinct, narrow, long, curved, distinctly crenulate.

***Wings***. Fore wing ~ 3.0× longer than maximum width. Pterostigma 3.2× longer than its maximum width. Radial vein (r) arising from distal 0.2 of pterostigma; radiomedial vein (2-SR) arising weakly behind middle of pterostigma and strongly separated from radial vein (r). Present only single and evenly curved abscissa of radial vein (r), which distinctly sclerotised in basal 0.25 and desclerotised on remaining part, reaching as track distal margin of wing. Radial (marginal) cell shortened, 3.2× longer than its maximum width. Sclerotised part of metacarp (1-R1) relatively long, faintly pigmented, 0.7× as long as pterostigma. First radiomedial vein (2-SR) sclerotised and brown, 4.5× longer than short recurrent vein (m-cu). Recurrent vein (m-cu) subinterstitial to first radiomedial vein (2-SR), approximately as long as sclerotised part of second medial abscissa (2-SR+M), posteriorly divergent with basal vein (1-M). Discoidal (discal) cell broadly sessile anteriorly, 1.5× longer than its maximum width. Nervulus (cu-a) perpendicular to longitudinal anal vein (1-1A), postfurcal, distance between basal vein (1-M) and nervulus (cu-a) 0.4× nervulus (cu-a) length. Hind wing ~ 4.0× longer than maximum width. First abscissa of mediocubital vein (M+CU) ~ 2.0× longer than second abscissa (1-M).

***Legs***. Hind coxa long, ~ 2.0× longer than maximum width. Hind femur 3.7× longer than maximum width. Hind tibia claviform, distinctly widened distally.

***Metasoma***. Metasoma approximately as long as mesosoma. All tergites distinctly sclerotised. First tergite with distinct and thick spiracular tubercles, strongly widened from base to spiracular tubercles situated almost in middle of tergite, then weakly narrowed towards posterior margin of tergite. First suture distinct, rather wide, curved, densely crenulate. Medial length of first tergite 0.4× its maximum width at level of spiracles, 0.7× as long as second tergite. Second suture present, but very fine and almost straight. Second tergite 1.3× longer than third tergite. Length of first to third tergites combined approximately equal to their maximum width. Third tergite straight on posterior margin.

***Sculpture***. Vertex densely and small punctate-reticulate, frons with additional granulation; face densely rugose-reticulate, below laterally almost smooth; clypeus finely rugulose to smooth in upper half and rugose-reticulate in lower half. Mesoscutum entirely densely punctate and smooth between punctulae, without very dense punctation in its medioposterior area. Scutellum widely smooth and with small sparse punctulae. Mesopleuron almost entirely smooth, with fine and sparse punctulae upper precoxal sulcus; metapleuron mainly smooth, narrowly areolate-rugose along its margins. Propodeum with fine submedial transverse keel, with narrow subtriangular areola delineated by carinae, and with wide and narrow petiolate area posteriorly; anterolateral areas distinctly delineated by carinae, almost smooth medially and reticulate marginally; propodeum mainly rugose-reticulate. First tergite entirely coarsely rugose-reticulate, with transverse subbasal carina. Second tergite mainly smooth, only rugose in basal 0.1. Remaining tergites entirely smooth.

***Colour***. Head reddish brown; mesosoma mainly dark reddish brown to black; metasoma reddish brown or faintly paler, its first tergite almost black. Antenna mainly dark brown, scape reddish brown. Palpi pale brown. Legs mainly yellow or pale brown, hind tibia and tarsus almost entirely dark reddish brown. Wing entirely infuscate, without dark bands. Pterostigma and parastigma brown, most veins of fore wing brown to pale brown.

**Female**. Unknown.

##### Distribution.

China (Liaoning Province).

#### ﻿ Paradelius (Sinadelius) ussuriensis

Taxon classificationAnimaliaHymenopteraBraconidae

Belokobylskij, 1988

C0A76591-1609-57F0-B702-9C2C8F42942E

Figs 11 , 12C

Paradelius ussuriensis Belokobylskij, 1988: 148; 1998: 556; Yu et al. 2016.

##### Material examined.

Russia. Primorskiy Territory: “Primorskiy Territory, 15 km S of Partizansk, forest, 16 VII 1979, Belokobylskij [col.]”, “Holotype *Paradeliusussuriensis* Belokobylskij”, 1 male (HT); Vladivostok, Sanatornaya, forest, 26.VII.1984 (S. Belokobylskij), 1 male (PT); 10 km S of Partizansk, forest, 19.VII.1979 (S. Belokobylskij), 1 male (PT); same locality, bushes, slopes, 11.VII.1996 (S. Belokobylskij), 2 males; 15 km S of Partizansk, forest, 17.VII.1979 (S. Belokobylskij), 1 male (PT); 10 km NW of Partizansk, forest, 13.VII.1979 (S. Belokobylskij), 1 male (PT); Lazovskiy Nature Reserve, 10 km SW of Sokolchi, 22 & 24.VII.1993 (S. Belokobylskij), 1 male; Spassk-Dal’niy, clearing, 13 & 17.VII.1991 (S. Belokobylskij), 1 female, 1 male; 20 km SE of Spassk-Dal’niy, forest, edge, 13 & 17.VII.1995 (S. Belokobylskij), 1 female, 1 male; 50 km N of Ol’ga, mixed forest, 30.VIII.1979 (S. Belokobylskij), 1 male (PT) (All in ZISP).

##### Description.

**Female**. Body length 2.4–2.5 mm; fore wing length 2.0–2.1 mm.

***Head***. Head 1.8–2.0× wider than its medial length (dorsal view), 1.1–1.2× wider than mesoscutum. Occiput distinctly concave. Head behind eyes weakly convex in anterior half and roundly narrowed in posterior half; transverse diameter of eye 1.2–1.4× larger than length of temple (dorsal view). Ocelli arranged in triangle with base 1.3–1.5× its sides. POL 2.3–2.5× Od, 0.6–0.8× OOL. Eye 1.4–1.5× as high as broad. Malar space 0.6–0.8× basal width of mandible, 0.15–0.20× height of eye. Face almost flat, width of face 1.4–1.5× its median height, ~ 1.2× height of eye. Tentorial pits distinct, distance between pits 1.2–1.5× distance from pit to eye. Clypeus high and distinctly convex, its width 2.0–2.1× median height, 0.6–0.7× width of face; its ventral margin almost straight. Head distinctly roundly narrowed below eyes (front view).

***Antenna***. Antenna more than 20-segmented (missing apically), weakly thickened, weakly setiform, all its segments elongated. Scape 2.1–2.2× longer than wide. First flagellar segment 3.0–3.3× longer than its apical width, 1.3–1.6× longer than second segment. Tenth segment ~ 1.3× longer than its maximum width.

***Mesosoma***. Mesosoma 1.4–1.5× longer than maximum height. Mesoscutum highly and convex-curvedly elevated above pronotum (lateral view), 1.5–1.7× as wide as its medial length (dorsal view). Prescutellar depression (scutellar sulcus) shallow, with distinct numerous carinae. Scutellum almost as long as anterior width. Prepectal carina not visible laterally. Precoxal sulcus distinct, narrow, short, curved, situated in middle of mesopleuron, rugose-reticulate.

***Wings***. Fore wing 2.4–2.6× longer than maximum width. Pterostigma 2.1–2.3× longer than its maximum width. Radial vein (r) arising from distal 0.1–0.2 of pterostigma. Radiomedial vein (2-SR) arising almost from or weakly behind middle of pterostigma and strongly separated from radial vein (r). Present only single and evenly curved abscissa of radial vein (r), which weakly sclerotised in basal 0.2–0.3 and desclerotised on remaining part, reaching as track distal margin of wing. Radial (marginal) cell shortened, 2.8–3.0× longer than its maximum width. Sclerotised part of metacarp (1-R1) relatively short, faintly pigmen­ted, 0.5–0.6× as long as pterostigma. First radiomedial vein (2-SR) sclerotised, 3.6–4.5× longer than recurrent vein (m-cu). Recurrent vein (m-cu) postfurcal to first radiomedial vein (2-SR), approximately as long as sclerotised part of second medial abscissa (2-SR+M), posteriorly subparallel with basal vein (1-M). Discoidal (discal) cell broadly sessile anteriorly, 1.1–1.2× longer than its maximum width. Nervulus (cu-a) subperpendicular to longitudinal anal vein (1-1A), postfurcal, distance between basal vein (1-M) and nervulus (cu-a) 0.4–0.7× nervulus (cu-a) length. Hind wing 3.3× longer than maximum width. First abscissa of mediocubital vein (M+CU) ~ 2.0× longer than second abscissa (1-M).

***Legs***. Hind coxa long and high, 1.5–1.7× longer than maximum width. Hind femur 3.3–3.5× longer than maximum width. Hind tibia claviform, 4.6–5.3× longer than maximum width, 0.7–0.8× as wide as hind femur; longest inner tibial spur 0.5–0.7× hind basitarsus length. Hind tarsus approximately as long as hind tibia, its basitarsus 0.6–0.7× as long as second–fifth segments combined. Second segment of hind tarsus 0.4× as long as basitarsus, 1.1× longer than fifth segments (without pretarsus).

***Metasoma***. Metasoma approximately as long as mesosoma. All tergites distinctly sclerotised. First tergite with distinct and thick spiracular tubercles, strongly widened from base to spiracular tubercles in middle of tergite, then rather distinctly narrowed towards posterior margin of tergite. First suture rather distinct, narrow, curved, crenulate. Medial length of first tergite 0.5–0.7× its maximum width at level of spiracles, 0.7–0.8× as long as second tergite. Se­cond suture present, but very fine and almost straight. Second tergite 1.1–1.3× longer than third tergite. Length of first–third tergites combined ~ 1.1× their maximum width. Third tergite straight in posterior margin. Ovipositor sheaths thickened, short, 0.3–0.4× as long as first–third tergites combined.

***Sculpture***. Head densely rugose-reticulate with punctation partly, vertex additionally with transverse striation laterally; clypeus entirely in dense punctation and smooth between punctures. Mesoscutum mainly rather densely punctate and smooth between punctulae, very densely punctate in subcircular area in its medioposterior half. Scutellum widely smooth with sparse punctulae. Mesopleuron almost entirely smooth, sometimes partly with sparse punctation; metapleuron mainly smooth, narrow areolate-rugose along its margins. Propodeum without or with fine submedial transverse keel, without or with wide areola delineated by carinae, widely densely rugulose-reticulate. First tergite entirely coarsely rugose-reticulate, with transverse subbasal carina. Second tergite mainly smooth, rugose-striate in basal 0.2–0.5. Remaining tergites entirely smooth.

***Colour***. Body dark reddish brown to black, sometimes head anteriorly and around eye reddish brown, rarely metasoma laterally and ventrally reddish brown to pale reddish brown at least partly. Antenna mainly dark reddish brown, black in apical half, scape reddish brown. Palpi pale brown or pale reddish brown, darkened basally. Legs mainly yellow, hind tibia dorsally and hind tarsus reddish brown to dark reddish brown; hind tibial spurs pale brown. Wing faintly infuscate, without dark bands. Pterostigma and parastigma dark brown, most veins brown to pale brown.

**Male**. Body length 2.4–2.6 mm; fore wing length 2.1–2.2 mm. Transverse diameter of eye 1.2–1.3× larger than length of temple (dorsal view). Antenna 22–23-segmented, subsetiform, all segments elongated. Tenth segment 1.8–1.9× longer than its maximum width. Penultimate segment 1.8–2.2× longer than its width, 0.8–0.9× as long as apical segment. Propodeum without submedial transverse keel and without areas delineated by carinae. Hind femur 3.4–3.7× longer than maximum width. Hind tibia distinctly claviform, 4.5–4.8× longer than maximum width, ~ 0.9× as wide as hind femur. Medial length of first tergite 0.6–0.7× its maximum width at level of spiracles, approximately as long as second tergite. Second tergite 1.3–1.5× longer than third tergite. Otherwise similar to female.

##### Distribution.

Russia (Primorskiy Territory).

## ﻿Discussion

The members of the tribe Adeliini are morphologically distinctly different from the other taxa of actual chelonine tribes: worldwide distributed Chelonini Foerster, 1863 and Phanerotomini Baker, 1926 (including Pseudophanerotomini Zettel, 1990), and Afrotropical Odontospaeropygini Zettel, 1990 (Zettel 1990; Yu et al. 2016; Kittel et al. 2016). One of the important diagnostic characters is located in the adeliine fore wing venation. Venation of the tribe Adeliini is very specialised, highly reduced, and includes several high-level valuable taxonomic characters, such as the second radiomedial vein (r-m) and second radiomedial (submarginal) cell are absent; the radial (marginal) cell distally is widely open; the radial (r) and first radiomedial (2-SR) veins are often separately arising from the pterostigma; the apical parts of the radial (r), medial (2-M), second longitudinal anal (2-1A), third cubital (3-CU1) and parallel (CU1a) veins are strongly reduced; the first transverse anal (2A) and brachial (CU1b) veins are completely absent and, as a result, the brachial (subdiscal) cell is widely open distally. In turn, in the members of the other three chelonine tribes, including the Phanerotomini molecularly the most related to Adeliini (Kittel et al. 2016), all discussed veins of the fore wing are present and distinctly sclerotised, and the cells are closed.

The structures of the metasoma are also seriously different between adeliines and other chelonines. In the members of the actual chelonine groups, the three immovably fused, heavily sclerotised and coarsely sculptured basal metasomal tergites are predominantly strongly enlarged and cover all following posterior segments; also a third tergite is often apically curved down and in some cases (many Chelonini) additionally below continued forwards. On the other side, all members of Adeliini have less sclerotised and often entirely or mainly smooth from first to third tergites, which are not covered the protruding far posterior segments; the third tergite never curved down posteriorly. The previously expressed opinion (Dowton and Austin 1998) about the plesiomorphic state of the carapace-like metasoma in the actual chelonines and derived (apomorphic) its condition in Adeliini is very questionable, because the evolutional transformation of such complicated structure of actual chelonine metasoma to much simple adeliine metasoma state is not easy to explain and justify. On the other hand, the characteristic for these taxonomic groups immovably fused the first to third tergites are also met in several genera from other Braconidae subfamilies (namely, Braconinae, Doryctinae, Rogadinae, Hormiinae, Telengaiinae, Acampsohelconinae, Brachistinae, etc.) (van Achterberg 1993), which showed the possibility of the parallel evolution of this character state in the different braconid phylogenetic lines and subfamilies.

Thus, discussed upper strong morphological differences are not allowed to definitely unite the members of adeliines with the representatives of the actual chelonines. Currently, the association of these groups inside the subfamily Cheloninae is supported mainly at the molecular level (Kittel et al. 2016). Nevertheless, a new molecular data based on the more number of genes will be very useful for the additional analyses of the phylogenetic relationship of these morphologically strongly different taxonomic groups.

## Supplementary Material

XML Treatment for Paradelius

XML Treatment for Paradelius (Paradelius) ghesquierei

XML Treatment for Paradelius (Paradelius) chinensis

XML Treatment for Subgenus Sculptomyriola

XML Treatment for Paradelius (Sculptomyriola) extremiorientalis

XML Treatment for Paradelius (Sculptomyriola) ghilarovi

XML Treatment for Paradelius (Sculptomyriola) koreanus

XML Treatment for Paradelius (Sculptomyriola) sinevi

XML Treatment for Subgenus Sinadelius

XML Treatment for Paradelius (Sinadelius) guangxiensis

XML Treatment for Paradelius (Sinadelius) nigricans

XML Treatment for Paradelius (Sinadelius) ussuriensis

## ﻿Additional information

### Author ORCIDs

Sergey A. Belokobylskij https://orcid.org/0000-0002-3646-3459

Deokseo Ku https://orcid.org/0000-0002-6274-6479

Xue-xin Chen https://orcid.org/0000-0002-9109-8853

### Data availability

All of the data that support the findings of this study are available in the main text.

## References

[B1] Belokobylskij SA (1988) Subfamily Adeliinae (Hymenoptera, Braconidae) in the Far East of the USSR. Proceeding of the All-Union Entomological Society 70: 144–152. [In Russian]

[B2] Belokobylskij SA (1998) Subfam. Adeliinae (Acaeliinae). In: Lehr PA (Ed.) Key to the insects of the Russian Far East.Vol. 4. Neuropteroidea, Mecoptera, Hymenoptera. Pt 3. Dal’nauka, Vladivostok, 553–558. [In Russian]

[B3] Belokobylskij SA Maetô K (2009) Doryctinae (Hymenoptera, Braconidae) of Japan. Fauna mundi. Vol. 1. Warszawska Drukarnia Naukowa, Warszawa, 806 pp.

[B4] Čapek M (1970) A new classification of the Braconidae (Hymenoptera) based on the cephalic structures of the final instar larva and biological evidence. Canadian Entomologist 102(7): 846–875. 10.4039/Ent102846-7

[B5] Chen XX van Achterberg C (2019) Systematics, phylogeny, and evolution of braconid wasps: 30 years of progress. Annual Review of Entomology 64(1): 335–358. 10.1146/annurev-ento-011118-111856 30332295

[B6] De Saeger H (1942) *Paradelius* genre nouveau de Microgastrinae (Hymenoptera: Braconidae). Revue de Zoologie et de Botanique Africaines 36: 313–316.

[B7] Dowton M Austin AD (1998) A phylogenetic relationships among the microgastroid wasps (Hymenoptera: Braconidae): combined analysis of 16S and 28S rDNA genes and morphological data. Molecular Phylogenetics and Evolution 10(3): 354–366. 10.1006/mpev.1998.0533 10051388

[B8] He JH Chen XX Ma Y (2000) Hymenoptera, Braconidae. Fauna Sinica. Insecta, Vol. 18. Science Press, Beijing, 757 pp.

[B9] Jasso-Martínez JM Santos BF Zaldívar-Riverón A Fernández-Triana JL Sharanowski BJ Richter R Dettman JR Blaimer BB Brady SG Kula RR (2022) Phylogenomics of braconid wasps (Hymenoptera, Braconidae) sheds light on classification and the evolution of parasitoid life history traits. Molecular Phylogenetics and Evolution 173: 107452. 10.1016/j.ympev.2022.107452 35307517

[B10] Kittel RN Austin AD Klopfstein S (2016) Molecular and morphological phylogenetics of chelonine parasitoid wasps (Hymenoptera: Braconidae), with a critical assessment of divergence time estimations. Molecular Phylogenetics and Evolution 101: 224–241. 10.1016/j.ympev.2016.05.016 27179700

[B11] Ku DS Belokobylskij SA Cha JY (2001) Hymenoptera (Braconidae). Economic Insects of Korea 16. Insecta Koreana. Supplement 23, 283 pp.

[B12] Muesebeck CFW Walkley LM (1951) Family Braconidae. In: Muesebeck CFW Krombein KV Townes HK (Eds) Hymenoptera of America North of Mexico. Synoptic catalog. U.S.Department, Agriculture Monograph 2: 90–184.

[B13] Quicke DLJ van Achterberg C (1990) Phylogeny of the subfamilies of the family Braconidae (Hymenoptera: Ichneumonoidea). Zoölogische Verhandelingen 258: 1–95.

[B14] Shi M Chen XX van Achterberg C (2005) Phylogenetic relationships among the Braconidae (Hymenoptera: Ichneumonoidea) inferred from partial 16S rDNA, 28S rDNA D2, 18S rDNA gene sequences and morphological characters. Molecular Phylogenetics and Evolution 37(1): 104–116. 10.1016/j.ympev.2005.03.035 15916906

[B15] Shimbori EM Bortoni MA Shaw SR Souza-Gessner CDS Cerântola Pde CM Penteado-Dias AM (2019) Revision of the New World genera *Adelius* Haliday and *Paradelius* de Saeger (Hymenoptera: Braconidae: Cheloninae: Adeliini). Zootaxa 4571(2): 151–200. 10.11646/zootaxa.4571.2.1 31715814

[B16] Tobias VI 1986. Subfam. Acaeliinae (Adeliinae). In: Medvedev GS (Ed.) Key to the insects of the USSR European part. Vol. 3. Hymenoptera. Pt 4. Nauka, Leningrad: 336–337. [In Russian]

[B17] van Achterberg C (1993) Illustrated key to the subfamilies of the Braconidae (Hymenoptera: Ichneumonoidea). Zoölogische Verhandelingen 283: 1–189.

[B18] Whitfield JB (1988) Two new species of *Paradelius* (Hymenoptera: Braconidae) for North America with biological notes. The Pan-Pacific Entomologist 64(4): 313–319.

[B19] Whitfield JB (1997) Adeliinae. In: Wharton RA Marsh PM Sharkey MJ (Eds) Manual of the New World genera of the family Braconidae (Hymenoptera). International Society of Hymenopterists. Special Publication No.1: 65–68.

[B20] Whitfield JB Mason WRM (1994) Mendesellinae, a new subfamily of braconid wasps (Hymenoptera, Braconidae) with a review of relationships within the microgastroid assemblage. Systematic Entomology 19(1): 61–76. 10.1111/j.1365-3113.1994.tb00579.x

[B21] Yu DS van Achterberg C Horstmann K (2016) Taxapad 2016. Ichneumonoidea 2015. Nepean, Ottawa, Ontario. [Database on flash-drive]

[B22] Zettel H (1990) Eine Revision der Gattungen der Cheloninae (Hymenoptera, Braconidae) mit Beschreibungen neuer Gattungen und Arten. Annalen des Naturhistorischen Museums in Wien 91B: 147–196.

